# Describing polyhedral tilings and higher dimensional polytopes by sequence of their two-dimensional components

**DOI:** 10.1038/srep40269

**Published:** 2017-01-17

**Authors:** Kengo Nishio, Takehide Miyazaki

**Affiliations:** 1 National Institute of Advanced Industrial Science and Technology (AIST), Central 2, Umezono 1-1-1, Tsukuba, Ibaraki 305-8568, Japan

## Abstract

Polyhedral tilings are often used to represent structures such as atoms in materials, grains in crystals, foams, galaxies in the universe, etc. In the previous paper, we have developed a theory to convert a way of how polyhedra are arranged to form a polyhedral tiling into a codeword (series of numbers) from which the original structure can be recovered. The previous theory is based on the idea of forming a polyhedral tiling by gluing together polyhedra face to face. In this paper, we show that the codeword contains redundant digits not needed for recovering the original structure, and develop a theory to reduce the redundancy. For this purpose, instead of polyhedra, we regard two-dimensional regions shared by faces of adjacent polyhedra as building blocks of a polyhedral tiling. Using the present method, the same information is represented by a shorter codeword whose length is reduced by up to the half of the original one. Shorter codewords are easier to handle for both humans and computers, and thus more useful to describe polyhedral tilings. By generalizing the idea of assembling two-dimensional components to higher dimensional polytopes, we develop a unified theory to represent polyhedral tilings and polytopes of different dimensions in the same light.

Partitioning a space with points into polyhedra in such a way that each polyhedron encloses exactly one point and then characterizing the polyhedral tiling is a promising strategy to study a wide range of structures^1^,^2^,^3^,^4^,^5^,^6^,^7^,^8^,^9^,^10^,^11^,^12^. For example, in studying the atomic structure of a material, the space can be divided into the so-called Voronoi polyhedra^1^,^2^,^3^,^4^,^5^,^6^,^7^,^8^,^9^, where each polyhedron encloses its associated atom. By using this method, for example, a way of how an atom *X* is surrounded by its first and second nearest-neighbour atoms is represented by the local tiling structure composed of the Voronoi polyhedra associated with the atom *X* and its first nearest-neighbour atoms.

Since such a local tiling structure can be regarded as a part of a four-dimensional polytope (4-polytope) called a polychoron, a method to describe how a polychoron is constructed from its building-block polyhedra can be used to study the structure of materials. For this reason, we have recently developed a theory of polytopes^13^ that is based on the hierarchy of structures of polytopes^14^,^15^,^16^,^17^,^18^: a polyhedron (3-polytope) is a tiling by polygons (2-polytopes), a polychoron (4-polytope) is a tiling by polyhedra (3-polytopes), and so on. Specifically, we have first created the *p*_3_*-*code for representing polyhedra. The *p*_3_*-*code consists of (1) an encoding algorithm for converting a way of how polygons are arranged to form a polyhedron into a *p*_3_*-*codeword (*p*_3_ for short) and (2) a decoding algorithm for recovering the original polyhedron from its *p*_3_. By generalizing the *p*_3_*-*code, we have created the *p*_4_*-*code for representing polychora. By using the *p*_4_*-*code, a way of how polyhedra are arranged to form a polychoron can be converted into a *p*_4_*-*codeword (*p*_4_ for short), from which the original polychoron can be recovered. A polyhedral tiling can be characterized by distribution of *p*_4_s of local tiling structures of different central polyhedra. However, *p*_4_ is redundant as described below.

The *p*_4_-codeword contains *p*_3_(1), *p*_3_(2), *p*_3_(3), 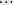 , and *p*_3_(*C*), where *p*_3_(*i*) is *p*_3_ of the polyhedron *i* and *C* is the number of polyhedra of the polychoron. Each *p*_3_(*i*) contains *p*_2_(*i*_1_), *p*_2_(*i*_2_), *p*_2_(*i*_3_), 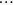, and *p*_2_(*i*_*F*(*i*)_), where each *p*_2_(*i*_*j*_) is the number of edges of the face *j* of the polyhedron *i* and *F*(*i*) is the number of faces of the polyhedron *i*. Here, we point out that *p*_2_(*i*_*j*_)s of all the faces of all the polyhedra are recorded in *p*_4_. However, since polyhedra are glued together face to face, the pair of faces glued each other have the same number of edges. *p*_4_ is thus redundant and lengthy. For example, if the face *y* of the polyhedron *x* is glued to the face *w* of the polyhedron *v*, then *p*_2_(*v*_*w*_) = *p*_2_(*x*_*y*_), so that *p*_2_(*v*_*w*_) in *p*_4_ is redundant.

Redundant codewords mean the lack of knowledge of structures of polychora. In addition, redundant codewords are practically unfavourable for both humans and computers. For humans, recognizing and writing down lengthy codewords are troublesome. For computers, larger hard drives are necessary to store codewords and more computation time is necessary to determine the equivalence of codewords.

In this paper, we develop a theory to reduce the redundancy in *p*_4_. For this purpose, we exploit the fact that the polyhedra are glued together face to face. Specifically, we regard two-dimensional regions shared by faces of adjacent polyhedra as building blocks of a polychoron. To distinguish between parts of a polychoron and parts of a polyhedra, we refer the two-dimensional building blocks of a polychoron to *ridges*. As the distinction between edges of a polyhedron and sides of a polygon was crucial for L. Euler to find his famous polyhedral formula, *V* − *E* + *F* = 2^14^, the distinction between ridges of a polychoron and faces of a polyhedron is crucial for our theory. To represent a polychoron using ridges, we formulate a method to convert *p*_4_ into 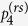, where the superscript “*rs*” indicates the *ridge-sequence*. Note that *p*_4_ instructs how to construct a polychoron from its building-block polyhedra, while 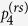 instructs how to construct a polychoron from its building-block ridges. The length of 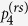 is as short as half of *p*_4_. By generalizing the method to higher-dimensional polytopes, we develop a unified theory of how a polytope is constructed from its two-dimensional components.

## Results

### Bare essentials of the *p*_4_-code

We will formulate the 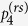-code consisting of (1) an encoding algorithm for converting *p*_4_ into 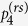 and (2) an decoding algorithm for recovering the original polychoron or *p*_4_ from 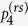. We start with the brief explanation of bare essentials of the *p*_4_-code needed to formulate the 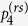-code. Specifically, we explain how to recover a polychoron from *p*_4_. For reader’s convenience, the encoding algorithm is described in Supplementary Note. The full details of the *p*_4_-code has been given in the previous paper^13^. Since polychora associated with disordered structures are simple, we deal with simple polychora. By a simple polychoron, we mean a polychoron whose 0-faces are all incident with four peaks. Here, 0-faces and peaks are zero- and one-dimensional components of a polychoron, respectively. Since a simple polychoron is composed of simple polyhedra, we first explain the *p*_3_-code for simple polyhedra. By a simple polyhedron, we mean a polyhedron whose vertices are all incident with three edges.

A polyhedron can be regarded as a tiling by polygons of the surface of a three-dimensional object that is topologically the same as a sphere. According to the idea developed by L. Euler, A. M. Legendre, F. Möbius, and P. R. Cromwell^14^, we assume that polygons are glued such that (1) any pair of polygons meet only at their sides or corners and that (2) each side of each polygon meets exactly one other polygon along an edge. We stress that we distinguish between parts of a polyhedron and those of the building-block polygons. Specifically, vertices and edges are zero- and one-dimensional parts of a polyhedron, respectively. On the other hands, corners and sides are zero- and one-dimensional parts of a polygon, respectively. Since this idea plays a central role in our theory, we need a verb to briefly describe the relation between parts of a polyhedron and those of polygons. For this purpose, we use the verb “*contribute*”. For example, when we say that the corners contribute to the vertex or the vertex is contributed by the corners, we mean that the vertex is a point on a polyhedron at which the corners of polygons meet. We also say that a polygon (side) contributes to a vertex if one of its corners (endpoints) contributes to the vertex. Similarly, when we say that the edge is contributed by the sides, we mean that the edge is a line segment on a polyhedron along which the sides of polygons meet. The face of a polyhedron is a polygon. But when we call a polygon, we regard it as a building block of a polyhedron. So, we may say the edge of a face. But we cannot say the edge of a polygon.

Using the *p*_3_-code, a way of how polygons are arranged to form a polyhedron can be converted into *p*_3_, which instructs how to construct the polyhedron from its building-block polygons. The *p*_3_-codeword consists of the polygon-sequence codeword (*ps*_2_) and the side-pairing codeword (*sp*), and is denoted as


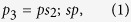


where “;” is a separator. The *ps*_2_-codeword is denoted as


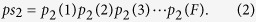


Here, *p*_2_(*i*) is the number of sides of the polygon *i,* and *F* is the number of polygons of the polyhedron. We note that the number of sides of the polygon *i* is identical with the number of edges of the face *i*.

If we know all information of *p*_2_(*i*)s and all information about which side should be glued to which side, we can construct a polyhedron by gluing polygons side to side. The *ps*_2_-codeword contains not only all information of *p*_2_(*i*)s, but also all or almost all information about which side should be glued to which side. Many polyhedra are represented just by *ps*_2_, but there are some polyhedra that need additional information about which side should be glued to which side. Such additional information is recorded in *sp*, which is denoted as


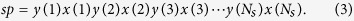


Here, *y*(*i*) and *x*(*i*) are the identification numbers (IDs) of sides. The pair of sides *y*(*i*) and *x*(*i*) is what we call a *non-curable additional pair (side-na-pair y*(*i*)*x*(*i*)). By a side-na-pair *y*(*i*)*x*(*i*), we mean that the sides *y*(*i*) and *x*(*i*) should be glued together. Here, *y*(*i*) > *x*(*i*) and *y*(*i*) < *y*(*i* + 1). *N*_s_ is the number of side-na-pairs.

Decoding *p*_3_ is constructing its original polyhedron by gluing together polygons side to side. To instruct which side should be glued to which side, we assign IDs to sides. We assign *i*_*j*_ to the *j*th side of the polygon *i*, and the side-ID *i*_*j*_ represents an integer: 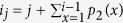. In constructing a polyhedron, if a side of a polygon of the partial polyhedron is not glued to the other polygon, we call it a *dangling side*. We abbreviate the smallest-ID dangling side as the *s-side*. We regard that an isolated corner as well as two corners meeting at a point forms a vertex of a partial polyhedron. We also regard that a dangling side forms an edge. If the pair of dangling sides contribute to a vertex that is also contributed by three polygons, that vertex is said to be *illegal*. When an illegal vertex (i-vertex) is generated, we *rectify* it by gluing together the two dangling sides contributing to it. The polyhedron can be recovered from *ps*_2_;*sp* as follows:

1.  (a)    The polygon 1 is a *p*_2_(1)-gon.

    (b)    Assign IDs 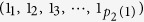 to its sides in a clockwise (CW) direction.

2.  (a)    The next polygon *i* (2 ≤ *i* ≤ *F*) is a *p*_2_(*i*)-gon.

    (b)    Assign IDs 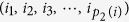 to its sides in a CW direction.

    (c)    Glue the side *i*_1_ to the s-side of the partial polyhedron.

    (d)    If *y*(*n*) (1 ≤ *n* ≤ *N*_s_) is the side ID of the polygon *i*, then glue the side *y*(*n*) to the side *x*(*n*) of the partial polyhedron.

    (e)    If i-vertices are generated, then rectify them, and repeat this procedure until no i-vertices remain.

3.  (a)    Repeat the procedure 2 until all polygons are placed.

The edge IDs are assigned as follows. Given that each edge is contributed by two sides, we tentatively assign the smaller side ID to the edge, and then relabel IDs so that the edge *i* is the one with the *i*th smallest tentative ID.

We note that the *p*_3_-code can be used to represent a tiling by polygons of a torus without modification. But to represent a toroidal polyhedron, we need to specify how to embed the torus in the 3-dimensional Euclidean space to form a toroidal polyhedron. The *p*_3_-code can also be generalized to non-orientable planes such as the Klein bottle^19^ by defining the clockwise direction for the polygon *i*, in which IDs are assigned to sides, depending on the clockwise direction for the polygon to which the side *i*_1_ is glued.

The *p*_3_-code is generalized to the *p*_4_-code for polychora as follows. We regard a polychoron as a tiling by polyhedra of the surface of a four-dimensional object that is topologically the same as a 3-sphere. We assume that polyhedra are glued together such that (1) any pair of polyhedra meet only at their faces, edges, or vertices and that (2) each face of each polyhedron meets exactly one other polyhedron along a ridge. We distinguish parts of a polychoron and parts of its building-block polyhedra. The 0-face, peak, and ridge are a point, line segment, and area on a polychoron, where the vertices, edges, and faces of polyhedra meet, respectively. The cell of a polychoron is a polyhedron.

The *p*_4_-codeword consists of a polyhedron-sequence codeword (*ps*_3_) and a face-pairing codeword (*fp*), and is denoted as


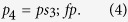


Here,


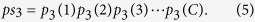


*C* is the number of polyhedra of the polychoron. *p*_3_(*i*) = *ps*_2_(*i*);*sp*(*i*) is *p*_3_ of the polyhedron *i*.

The *fp*-codeword consists of what we call *face-na-pairs wzv*, and is denoted as


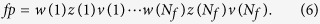


Here, *w*(*i*) and *v*(*i*) are face IDs. *w*(*i*) > *v*(*i*) and *w*(*i*) < *w*(*i* + 1). *z*(*i*) is the global side ID of a side of the polygon *w*(*i*). The global side IDs will be explained latter. By a face-na-pair *w*(*i*)*z*(*i*)*v*(*i*), we mean that the faces *w*(*i*) and *v*(*i*) should be glued together in such a way that the edge of the face *w*(*i*) contributed by the side *z*(*i*) is glued to the smallest-ID edge of the face *v*(*i*). *N*_f_ is the number of face-na-pairs of the polychoron. Note that, in order to formulate 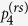, *fp* of the present work is slightly modified from the original definition^13^. For the original definition, *z*(*i*) is the edge ID of the edge of the face *w*(*i*) glued to the smallest-ID edge of the face *v*(*i*).

In decoding *p*_4_, if a face of a polyhedron of the partial polychoron is not glued to the other face, we call it a *dangling face*. By the edge *i*_*j*_ (face *i*_*j*_), we mean the *j*th edge (face) of the polyhedron *i*. We abbreviate the smallest-ID dangling face as the *s-face*. We regard that an isolated edge as well as two edges meeting along a line segment forms a peak of a partial polychoron. We also regard that a dangling face forms a ridge. In a partial polychoron, if the pair of dangling faces contribute to a peak that is also contributed by three polyhedra, we call that peak an *illegal peak (i-peak*). When an i-peak is generated, we rectify it by gluing together the two dangling faces contributing to it. The polychoron can be recovered from *ps*_3_;*fp* as follows:

1.  (a)   Decode *p*_3_(1) to obtain the polyhedron 1, assigning face and edge IDs.

2.  (a)    Decode *p*_3_(*i*) to obtain the next polyhedron *i* (2 ≤ *i* ≤ *C*), assigning face and edge IDs.

    (b)    Glue the face *i*_1_ to the s-face of the partial polychoron in such a way that the edge *i*_1_ is glued to the smallest-ID edge of the s-face.

    (c)    If *w*(*n*) (1 ≤ *n* ≤ *N*_*f*_) is the face ID of the polyhedron *i*, then glue the face *w*(*n*) to the face *v*(*n*) of the partial polyhedron in such a way that the edge of the face *w*(*n*) contributed by the side *z*(*n*) is glued to the smallest-ID edge of the face *v*(*n*).

    (d)    If i-peaks are generated, then rectify them, and repeat this procedure until no i-peaks remain.

3.  (a)    Repeat the procedure 2 until all polyhedra are placed.

Ridge IDs are assigned as follows. Given that each ridge is contributed by two faces, we tentatively assign the smaller face ID to the ridge. We call the ID thus assigned the *tentative ridge ID*. We then relabel IDs so that the ridge *i* is the one with the *i*th smallest tentative ID. The tentative ridge IDs and ridge IDs play a key role in reducing the redundancy in *p*_4_. Peak IDs are also assigned similarly.

### Preliminary arrangements

**Figure 1:**
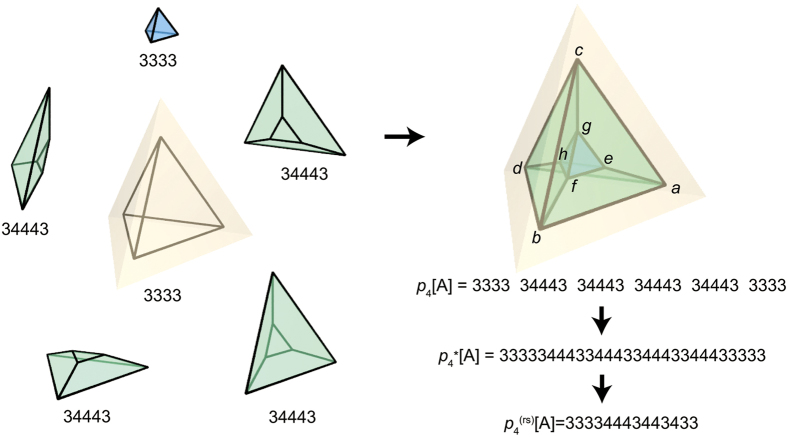
Overview of the 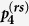-code. Three-dimensional Schlegel diagrams^13^,^15^,^18^ (a projection from four- to three-dimensional space) are used to illustrate the polychoron. Note that the interior of the polyhedron *abcd* on the polychoron in four-dimensional space (not shown) is mapped to the exterior of the outside polyhedron *abcd* on the Schlegel diagram.

An outline of converting *p*_4_ into 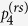 is illustrated in Fig. 1. We will first break *p*_4_ down into its *ps*_2_s and *sp*s, and reconstruct 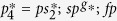, which provides us a good perspective for reducing the redundancy. We will then reduce the redundancy by converting 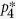 into 
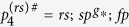
. Finally, to make our theory more beautiful, we unify *sp*^g*^ and *fp* into a *part-pairing* codeword (*pp*), and obtain 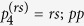.

To formulate 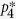, we distinguish *local* IDs and *global* IDs. When we call the polygon *j* of the polyhedron *i*, the number *j* is what we call the local polygon ID associated with the polyhedron *i*. We can designate the same polygon as the polygon *i*_*j*_. The symbol *i*_*j*_ is what we call the global polygon ID of the polychoron. The symbol *i*_*j*_ also represents the number: 
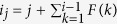
, where *F*(*k*) is the number of polygons of the polyhedron *k*. Similarly, by the side *m*_*n*_ of the polyhedron *i*, we mean the *n*th side of the polygon *m* of the polyhedron *i*. The number *n* is the local side ID associated with the polygon *m* of the polyhedron *i*, while the symbol *m*_*n*_ is the local side ID associated with the polyhedron *i* and is also the number, 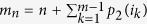. Here, *p*_2_(*i*_*k*_) is the number of sides of the polygon *i*_*k*_. Using the global side ID, we can designate the side *m*_*n*_ of the polyhedron *i* as the side 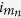. The symbol 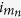 also represents the number: 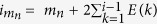, where *E*(*k*) is the number of edges of the polyhedron *k*.

The *sp*(*i*)-codeword of *p*_3_(*i*) = *ps*_2_(*i*);*sp*(*i*) is written using the local side IDs associated with the polyhedron *i*. Using the global side IDs, we rewrite *p*_3_(*i*) as 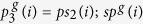. Here, 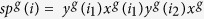

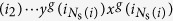
 is obtained by translating 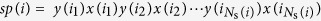 from local side ID into global side ID. 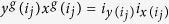. *y*(*i*_*j*_) and *x*(*i*_*j*_) are the local side IDs for the *j*th side-na-pair of the polyhedron *i. N*_s_(*i*) is the number of side-na-pairs of the polyhedron *i*. For example, *p*_3_(*i*) = 46565475543; 7_4_4_4_ = 46565475543;34 19 is rewritten as 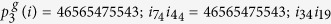. Reversely, *p*_3_(*i*) can be recovered from 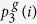 just by translating the global side IDs *i*_*y*_*i*_*x*_ into their corresponding local side IDs *yx*.

By putting together *sp*^*g*^(*i*)s, *sp*^g*^ is defined as follows:


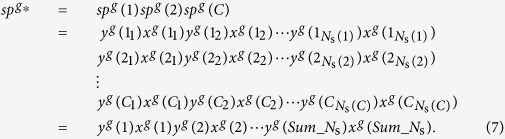


Here, 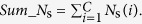


Similarly, 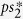 is defined by putting together *ps*_2_(*i*)s as follows:


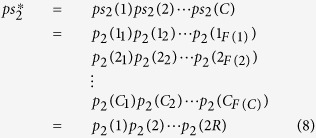


In the last transformation, we translated the symbols *i*_*j*_ into the corresponding numbers. *R* is the number of ridges of the polychoron.

By putting 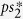, *sp*^g*^, and *fp* together, 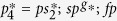. For example, for a polychoron *A* shown in Fig. 1, 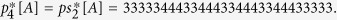


We can recover *p*_4_ from 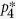 as follows. By construction, the first *F*(1) digits of 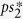 form *ps*_2_(1). However, we do not know *F*(1) beforehand. To find out *F*(1), we regard 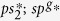 as *p*_3_, and decode it until a polyhedron is completed. Suppose that a polyhedron is completed when the *α*th digit of 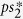 is decoded. Then *F*(1) = *α*, and 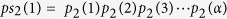. If the sides of the polyhedron are found in *sp*^g*^, record them in *sp*^*g*^(1). We then remove *ps*_2_(1) from 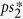, and obtain 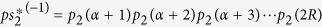. As with 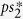, the first *F*(2) digits of 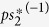 form *ps*_2_(2). To find out *F*(2), we decode 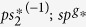 using the *p*_3_-code. Suppose that a polyhedron is completed when the *β*th digit of 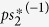 is decoded. Then *F*(2) = *β*, and 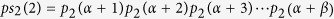. If the sides of the polyhedron are found in *sp*^g*^, record them in *sp*^*g*^(2). We then remove *ps*_2_(2) from 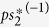, and obtain 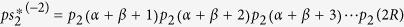. By repeating this procedure, we can determine 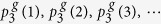, and therefore *p*_4_. As an example, we illustrate the procedures of recovering *p*_4_[*A*] from 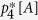 in Supplementary Note.

### Reveal and remove redundancy in 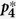


To reveal the redundancy in 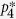, we observe how the polychoron *A* shown in Fig. 1 is recovered from 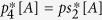. After determining *p*_4_[*A*], we construct the polyhedron 1 (3333) and polyhedron 2 (34443), and then glue them together in such a way that the face 2_1_ is glued to the face 1_1_. Therefore, *p*_2_(2_1_) must be equal to *p*_2_(1_1_). Thus, *p*_2_(2_1_) is redundant. Next we attach the polyhedron 3 (34443) to the partial polychoron in such a way that the face 3_1_ is glued to the face 1_2_ of the partial polychoron. Therefore, *p*_2_(3_1_) must be equal to *p*_2_(1_2_), and *p*_2_(3_1_) is redundant. In addition, to rectify an i-peak, we glue together the faces 3_2_ and 2_2_. Therefore, *p*_2_(3_2_) must be equal to *p*_2_(2_2_), and *p*_2_(3_2_) is redundant.

In general, when two faces *i*_*j*_ and *m*_*n*_ (*m* > *i*) are glued together, *p*_2_(*m*_*n*_) is redundant, while *p*_2_(*i*_*j*_) is essential. Since the face *m*_*n*_ meets the face *i*_*j*_ along the ridge with the tentative ID *i*_*j*_, *p*_2_(*m*_*n*_) = *p*_2_(*i*_*j*_) = *r*_*t*_(*i*_*j*_). Here, *r*_*t*_(*x*_*y*_) is the number of peaks of the ridge with the tentative ID *x*_*y*_. Thus, the number of peaks of every ridge is doubly recorded in 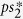.

Returning to the polyhedron *A*, we will remove all the redundant *p*_2_(*m*_*n*_)s from 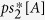 and construct the sequence of essential *p*_2_(*i*_*j*_)s,


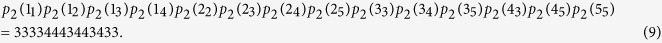


The sequence of essential *p*_2_(*i*_*j*_)s is identical with the sequence of *r*_*t*_(*i*_*j*_)s,


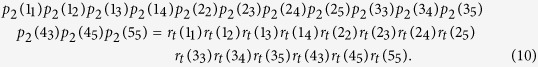


By rewriting the sequence using the ridge IDs (not tentative ridge IDs), we obtain what we call the ridge-sequence codeword (*rs*),


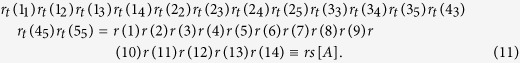


Here, *r*(*i*) is the number of peaks of the ridge with the ID *i*. The number of peaks of every ridge is recorded just once in *rs*, and the redundancy is thus reduced. In general, the redundancy can be reduced by modifying *p*_4_ into 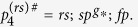 Here, *rs* = *r*(1)*r*(2)*r*(3) 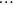 *r*(*R*).

### How to recover p^4^ from 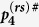


**Figure 2:**
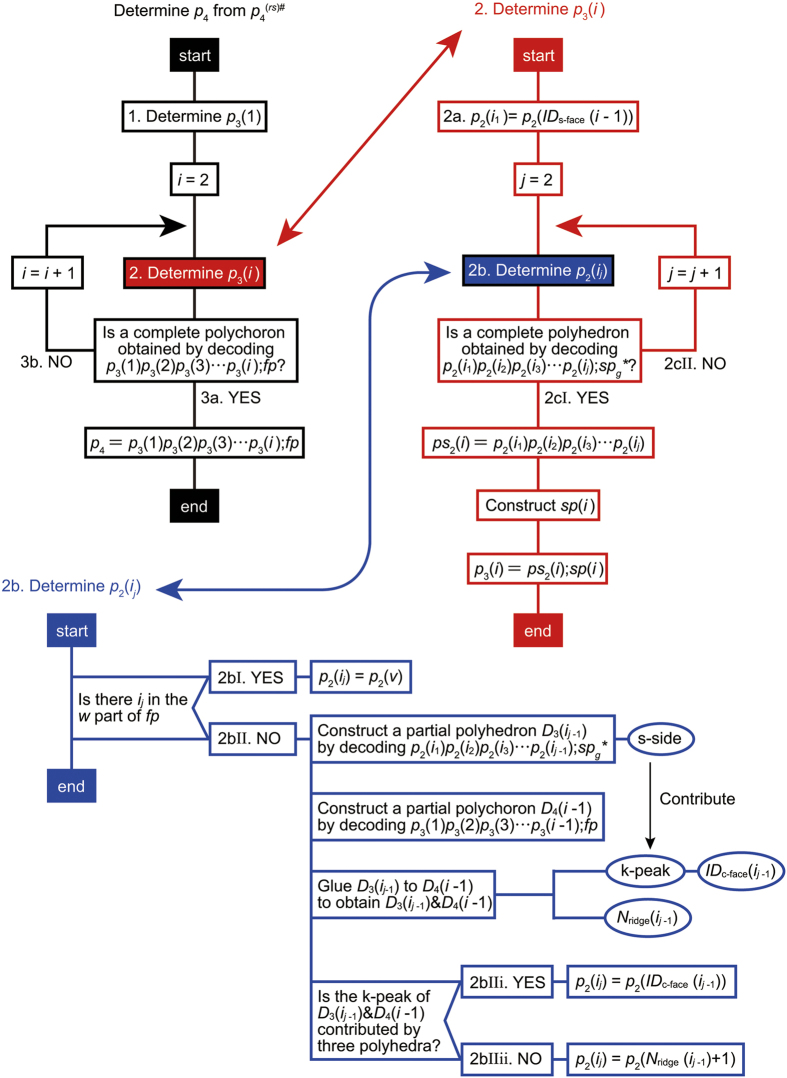
Procedures for recovering *p*_4_ from 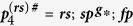.

The 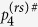-codeword contains information about how to assemble ridges to form a polychoron in the sense that *p*_4_ can be recovered from 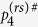. As is summarized in Fig. 2, to recover *p*_4_, we determine 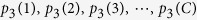 step-by-step. To determine *p*_3_(*i*), we deduce 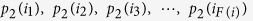 step-by-step. To deduce *p*_2_(*i*_*j*_), we examine whether the face *i*_*j*_ should be glued to an existing face of the partial polychoron or create a new ridge.

We first describe how to determine *p*_3_(1) = *ps*_2_(1);*sp*(1) from 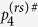. All faces of polyhedron 1 create new ridges. By construction, the first *F*(1) digits of *r*(1)*r*(2)*r*(3) 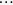 *r*(*R*) form *ps*_2_(1). However, we do not know *F*(1) beforehand. To find out *F*(1), we regard *r*(1)*r*(2)*r*(3) 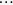 *r*(*R*);*sp*^g*^ as *p*_3_, and decode it until a polyhedron is completed. The polyhedron thus obtained is the polyhedron 1. Suppose that a polyhedron is completed when the *α*th face is decoded. Then *F*(1) = *α*, and *ps*_2_(1) = *r*(1)*r*(2)*r*(3) 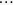 *r*(*α*). Every time we recover a polygon in the decoding process, we search *sp*^g*^ for side-na-pairs of the polyhedron 1. If side-na-pairs are found, we record their corresponding local side IDs in *sp*(1). By combining *ps*_2_(1) and *sp*(1), we obtain *p*_3_(1) = *ps*_2_(1);*sp*(1).

For 2 ≤ *i, p*_3_(*i*) can be determined from 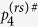 and *p*_3_(1)*p*_3_(2)*p*_3_(3) 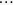 *p*_3_(*i* − 1). Our first task is to deduce *p*_2_(*i*_1_). For this purpose, we construct a partial polychoron *D*_4_(*i* − 1), which is obtained by decoding *p*_3_(1)*p*_3_(2)*p*_3_(3) 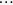 *p*_3_(*i* − 1);*fp*. Since the face 1 of the polyhedron *i* should be glued to the s-face of *D*_4_(*i* − 1), *p*_2_(*i*_1_) = *p*_2_(*ID*_s−face_(*i* − 1)). Here, *ID*_s−face_(*k*) is the global face ID of the s-face of *D*_4_(*k*).

For 2 ≤ *j, p*_2_(*i*_*j*_) can be determined from 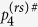, *p*_3_(1)*p*_3_(2)*p*_3_(3) 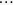 *p*_3_(*i* − 1), and *p*_2_(*i*_1_)*p*_2_(*i*_2_)*p*_2_(*i*_3_) 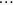 *p*_2_(*i*_*j*−1_). To deduce *p*_2_(*i*_*j*_), we examine whether the face *i*_*j*_ should be glued to an existing face or create a new ridge. We first search the *w* part of *fp* for *i*_*j*_. If *i*_*j*_ is found, the faces *i*_*j*_( = *w*) and *v* form a face-na-pair. Since those faces should be glued together, *p*_2_(*i*_*j*_) = *p*_2_(*v*). If *i*_*j*_ is not found, we construct a partial polyhedron *D*_3_(*i*_*j*−1_), which is obtained by decoding *p*_2_(*i*_1_)*p*_2_(*i*_2_)*p*_2_(*i*_3_) 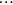 *p*_2_(*i*_*j*−1_);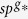 using the *p*_3_-code. We then glue the face *i*_1_ (of *D*_3_(*i*_*j*−1_)) to the s-face of *D*_4_(*i* − 1) in such a way that the edge *i*_1_ is glued to the smallest-ID edge of the s-face. If *w*(*n*) (1 ≤ *n* ≤ *N*_f_) is the face ID of *D*_3_(*i*_*j*−1_), then glue the face *w*(*n*) to the face *v*(*n*) of *D*_4_(*i* − 1) in such a way that the edge of the face *w*(*n*) contributed by the side *z*(*n*) is glued to the smallest-ID edge of the face *v*(*n*). If i-peaks are generated, then we rectify them until no i-peaks remain. We write *D*_3_(*i*_*j*−1_) & *D*_4_(*i* − 1) for the partial polychoron thus obtained. The peak contributed by the s-side of *D*_3_(*i*_*j*−1_) plays a key role in determining whether the face *i*_*j*_ should be glued to an existing face or create a new ridge, therefore we call that peak the *key peak (k-peak*). We write *ID*_k−peak_(*i*_*j*−1_) for the global peak ID of the k-peak. There exists one dangling face contributing to the k-peak, which we call a *candidate face (c-face*). We write *ID*_c−face_(*i*_*j*−1_) for the global face ID of the c-face. The face *i*_*j*_ will be glued to the face *ID*_c−face_(*i*_*j*−1_) or create a new ridge. Now, we need to consider two cases:

(case 1)     The k-peak of *D*_3_(*i*_*j*−1_) & *D*_4_(*i* − 1) is contributed by three polyhedra (*D*_3_(*i*_*j*−1_) and two from *D*_4_(*i* − 1)). In this case, in constructing *D*_3_(*i*_*j*_) & *D*_4_(*i* − 1), when the peak *ID*_k−peak_(*i*_*j*−1_) is contributed by three polyhedra and the face *i*_*j*_ is not glued to the face *ID*_c−face_(*i*_*j*−1_), that peak will be illegal. To rectify the i-peak, the faces *i*_*j*_ and *ID*_c−face_(*i*_*j*−1_) should be glued together. Thus, *p*_2_(*i*_*j*_) = *p*_2_(*ID*_c−face_(*i*_*j*−1_)).

(case 2)     The k-peak of *D*_3_(*i*_*j*−1_) & *D*_4_(*i* − 1) is contributed by two polyhedra (*D*_3_(*i*_*j*−1_) and one from *D*_4_(*i* − 1)). In this case, the face *i*_*j*_ should create a new ridge. Thus, *p*_2_(*i*_*j*_) = *r*(*N*_ridge_(*i*_*j*−1_) + 1). Here, *N*_ridge_(*m*_*n*_) is the number of ridges of *D*_3_(*m*_*n*_) & *D*_4_(*m* − 1).

### Part-pairing codeword and 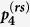


To make our theory more beautiful, we modify the way to record na-pairs. The side-na-pairs *yx* are recoded in *sp*^g*^, while the face-na-pairs *wzv* are in *fp*. To distinguish them, there is a separator “;” between *sp*^g*^ and *fp* as 
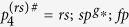
. To remove the separator, we will unify *sp*^g*^ and *fp* into *pp*. Finally, we will obtain 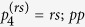. As a result, both polyhedra and polychora are represented by codewords of the same format, namely two number sequences separated by “;”.

To formulate *pp*, we introduce the notion of *parts*. We regard the sides of a polygon are parts of that polygon. We also regard the polygon itself is the part of that polygon. We define the set of parts of the polygon *i* as


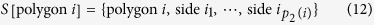


Similarly, for *j* > 2, we define the set of parts of the polyhedron *j* as


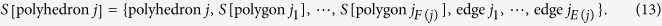


We assign IDs to parts such that we can identify side-na-pairs *yx* and face-na-pairs *wzv* in recovering the original polychoron. To meet this requirement, we assign IDs to parts of the polychoron in the order of *S*[polygon 1], polyhedron 1, *S*[polygon 2], …, *S*[polygon *F*(1)], edges of polyhedron 1, polychoron 1, *S*[polyhedron 2], …, *S*[polyhedron *C*], ridges of polychoron 1, peaks of polychoron 1.

The *pp*-codeword is obtained as follows. We first translate *sp*^g*^;*fp* into part ID. Then we remove the separator “;”. Finally, we obtain


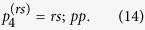


The side- and face-na-pairs can be identified from *pp* as follows. Let *p*(*i*) be the *i*th digit of *pp*. If the part *p*(*i*) is a side *Y*, the part *p*(*i* + 1) is a side *X*, and *Y* > *X*, then the pair *p*(*i*)*p*(*i* + 1) is a side-na-pair. If the part *p*(*i*) is a face *W* and the part *p*(*i* + 2) is a face *V*, then the pair *p*(*i*)*p*(*i* + 1)*p*(*i* + 2) is a face-na-pair.

Note that the amount of tasks needed to generate 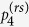 is comparable to that needed to generate *p*_4_. This is because converting *p*_4_ to 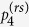 amounts to just assigning IDs to ridges and parts of the polychoron. We also note that the length of 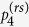 is shorter than that of *p*_4_ by R^*^, where R^*^ is the number of ridges contributed by two faces. R^*^ = *R* for a polychoron, while R^*^ < *R* for a partial polychoron. Therefore, the compression efficiency gets worse for partial polychora. As described above, by converting 3333 34443 34443 34443 34443 3333 into 33334443443433, data of the polychoron is compressed to half. On the other hand, for example, *p*_4_ and 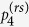 of a partial polychoron composed of one 3333-polyhedron and one 34443-polyhedron are 3333 34443 and 33334443, respectively. Just one number “3” is removed by converting *p*_4_ into 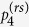.

**Figure 3:**
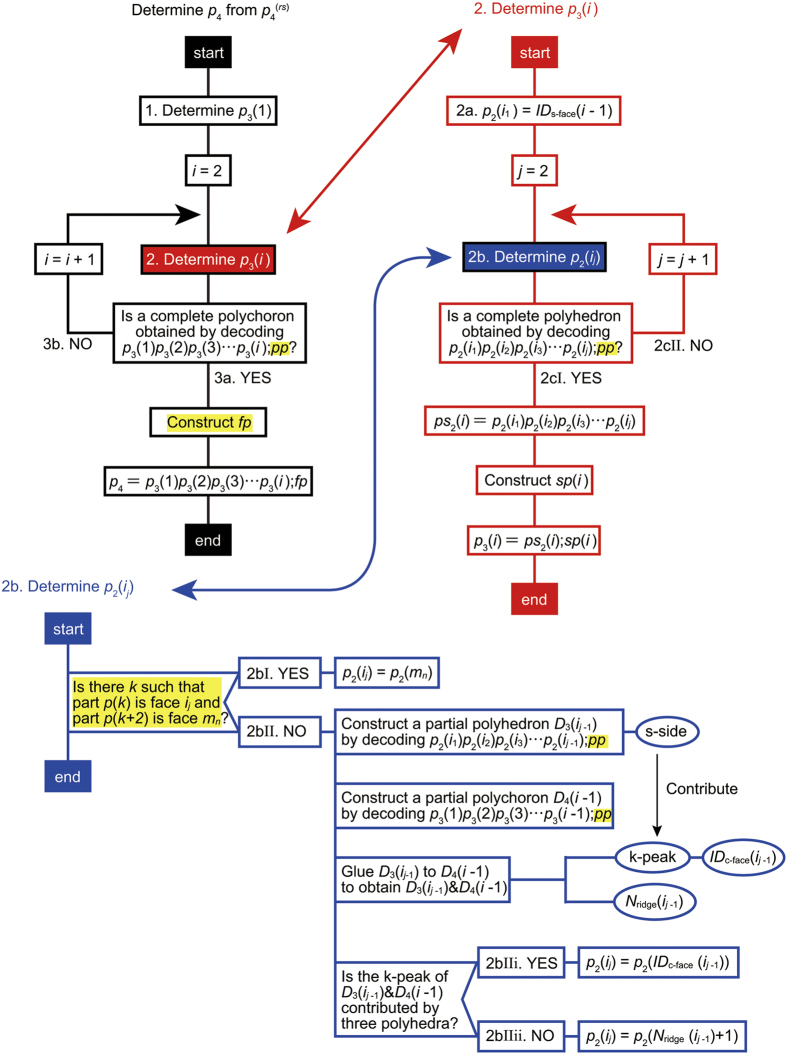
Procedures for recovering *p*_4_ from 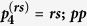. The differences from the algorithm for 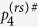 is highlighted in yellow.

The *p*_4_-codeword can be recovered from 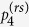 by modifying the procedure for recovering *p*_4_ from 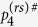 as follows (Fig. 3):

1. Determine *p*_3_(1) = *ps*_2_(1);*sp*(1) as follows:
  - Decode *r*(1)*r*(2)*r*(3) ··· *r*(*R*);*pp* using the the *p*_3_-code.
  - If a polyhedron is completed when the *α*th face is decoded, then *ps*_2_(1) = *r*(1)*r*(2)*r*(3) … *r*(*α*).
  - If side-na-pairs of the polyhedron are found in *pp*, record their corresponding local side IDs in *sp*(1).
2. Determine the next *p*_3_ = *ps*_2_(*i*);*sp*(*i*)(2 ≤ i) as follows:
  - *p*_2_(*i*_1_) = *p*_2_(*ID*_s-face_(*i* − 1)).
  - To determine the next *p*_2_(*i*_*j*_) (2 ≤ j), we search *pp* for the part ID of the face *i*_*j*_. Here, two cases arise:
    - If the part *p*(*k*) is the face *i*_*j*_ and the part *p*(*k* + 2) is a face, then let *m*_*n*_ be the face-ID of the part *p*(*k* + 2), and *p*_2_(*i*_*j*_) = *p*_2_(*m*_*n*_).
    - Otherwise, we examine the k-peak, and then additional two cases arise:
      - If the k-peak is contributed by three polyhedra, then *p*_2_(*i*_*j*_) = *p*_2_(*ID*_c-face_(*i*_*j*−1_)).
      - Otherwise, *p*_2_(*i*_*j*_) = *r*(*N*_ridge_(*i*_*j*−1_) + 1).
  - Decode *p*_2_(*i*_1_)*p*_2_(*i*_2_)*p*_2_(*i*_3_) … *p*_2_(*i*_*j*_);*pp* using the the *p*_3_-code. Two cases then arise:
    - If a polyhedron is completed, then *ps*_2_(*i*) = *p*_2_(*i*_1_)*p*_2_(*i*_2_)*p*_2_(*i*_3_) … *p*_2_(*i*_*j*_). If side-na-pairs of the polyhedron are found in *pp*, record their corresponding local side IDs in *sp*(*i*). *p*_3_(*i*) is thus determined.
    - Otherwise, repeat the procedure 2b.
3. Decode *p*_3_(1)*p*_3_(2)*p*_3_(3) … *p*_3_(*i*);*pp* using the the *p*_4_-code. Two cases then arise:
  - If a polychoron is completed, then *ps*_3_ = *p*_3_(1)*p*_3_(2)*p*_3_(3) 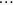 *p*_3_(*i*). If face-na-pairs are found in *pp*, record their corresponding global face, side, and face IDs in *fp*. Thus, *p*_4_ = *ps*_3_;*fp* is determined.
  - Otherwise, repeat the procedure 2.

**Figure 4:**
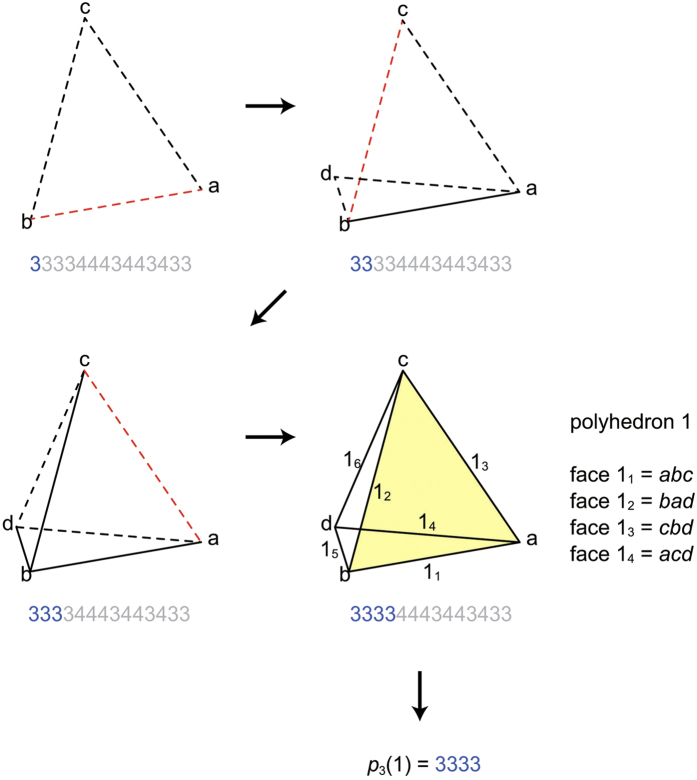
How to determine *p*_3_(1) from 33334443443433. The dashed lines are the edges contributed by one polygon. The solid lines are the edges contributed by two polygons. Each s-side to which the next polygon is glued is coloured red. For the completed polyhedron 1, global edge IDs are shown near their edges. The polyhedron 1 is *D*_4_(1), and its s-face is coloured yellow.

**Figure 5:**
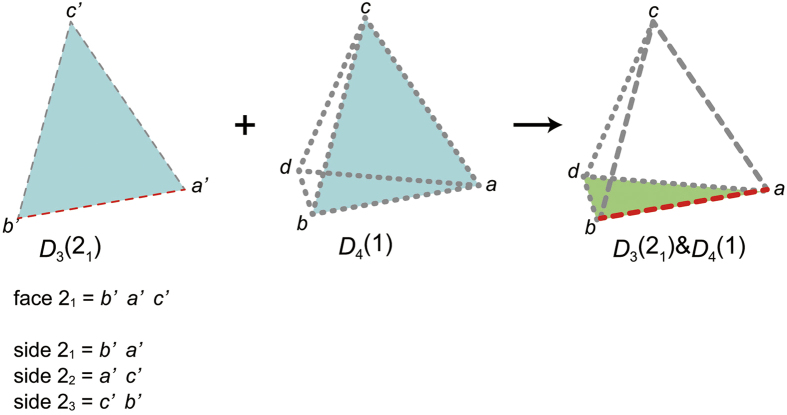
Partial polychoron *D*_*3*_(2_1_) & *D*_4_(1). *D*_3_(2_1_), *D*_4_(1), and *D*_*3*_(2_1_) & *D*_4_(1) are illustrated using three-dimensional Schlegel diagrams. The polyhedron *abcd* is the outside polyhedron. The dotted and dashed bold lines of the partial polychora indicate peaks contributed by one and two polyhedra, respectively. The face 2_1_ (of *D*_3_(2_1_)) and the s-face of *D*_4_(1) (face 1_1_) are coloured blue. By gluing together the blue faces, *D*_*3*_(2_1_) & *D*_4_(1) is obtained. The s-side *b*′*a*′ of *D*_3_(2_1_) (red dashed line) contributes to the peak *ab* of *D*_*3*_(2_1_) & *D*_4_(1) (red-bold-dashed line), so that the peak *ab* is the k-peak. The dangling face *bad* is the c-face, for it contributes to the k-peak. The c-face is coloured green. The k-peak is contributed by two polyhedra (polyhedron 1 and *D*_3_(2_1_)). Global face and side IDs of *D*_3_(2_1_) are shown near *D*_3_(2_1_) for reference. Note that since the polyhedron 2 is an inside polyhedron, a counter CW direction such as *b*′ → *a*′ → *c*′ around the face *b*′*a*′*c*′ of the polyhedron 2 on the Schlegel diagram corresponds to a CW direction around the corresponding face of the polychoron in four-dimensional space.

**Figure 6:**
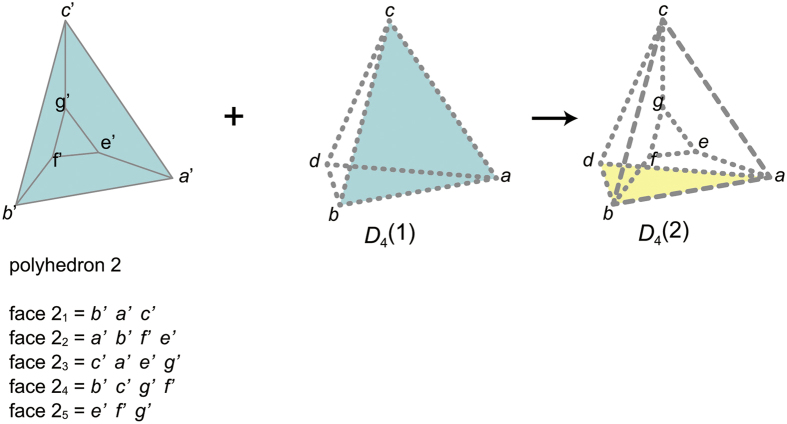
Partial polychoron *D*_4_(2). By gluing the blue face 2_1_ (of polyhedron 2) to the blue s-face of *D*_4_(1), *D*_4_(2) is obtained. The s-face of *D*_4_(2) (face 1_2_) is coloured yellow.

**Figure 7:**
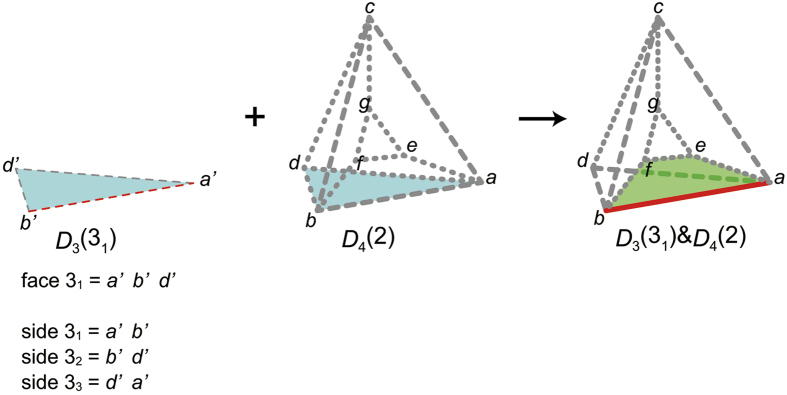
Partial polychoron *D*_3_(3_1_) & *D*4(2). By gluing the blue face 3_1_ (of *D*_3_(3_1_)) to the blue s-face of *D*_4_(2), D_4_(3_1_) & D_4_(2) is obtained. The red-bold-solid peak *ab* of D_4_(3_1_) & D_4_(2) is the k-peak, for it is contributed by the s-side *a*′*b*′ of *D*_3_(3_1_). The green dangling face 2_2_ is the c-face, for it contributes to the k-peak. Three polyhedra (polyhedra 1 and 2 and *D*_3_(3_1_)) contribute to the k-peak.

**Figure 8:**
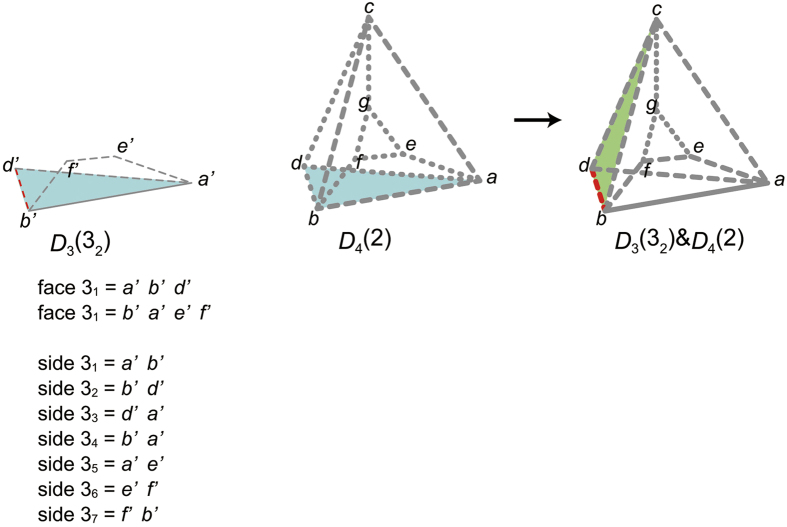
Partial polychoron *D*_3_(3_2_) & *D*4(2). The k-peak *bd* (red-bold-dashed line) is contributed by two polyhedra (polyhedron 1 and *D*_3_(3_2_)).

As an example, we illustrate how to recover *p*_4_[*A*] from 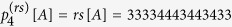 as follows:

1. We decode *rs*[*A*] using the *p*_3_-code. When the 4th digit is decoded, a 3333-polyhedron is obtained, thereby it turns out *p*_3_(1) = 3333 (Fig. 4).
2. We determine *p*_3_(2) as follows:
  - The 3333-polyhedron is the partial polychoron *D*_4_(1). The s-face of *D*_4_(1) is the face 1_1_ (Fig. 4). Since the face 1 of the polyhedron 2 will be glued to the face 1_1_, *p*_2_(2_1_) = *p*_2_(1_1_) = 3.
  - We construct the partial polyhedron *D*_3_(2_1_), glue it to the partial polychoron *D*_4_(1), and obtain the partial polychoron *D*_3_(2_1_) & *D*_4_(1) (Fig. 5). Since the k-peak *ab* is contributed by two polyhedra (polyhedron 1 and *D*_3_(2_1_)), the face 2_2_ will create a new ridge. Since 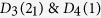 has four ridges *abc, bad, cbd*, and *acd, N*_ridge_(2_1_) = 4. Thus, *p*_2_(2_2_) = *r*(*N*_ridge_(2_1_) + 1) = *r*(5) = 4.
  - For the same reason, *p*_2_(2_3_) = *r*(6) = 4, *p*_2_(2_4_) = *r*(7) = 4, *p*_2_(2_5_) = *r*(8) = 3.
  - When we decode *p*_2_(2_1_)*p*_2_(2_2_)*p*_2_(2_3_)*p*_2_(2_4_)*p*_2_(2_5_) = 34443, a polyhedron is completed, thereby it turns out *p*_3_(2) = 34443.
3. We determine *p*_3_(3) as follows:
  - We construct *D*_4_(2) from the partial *p*_4_-codeword: *p*_3_(1)*p*_3_(2) = 3333 34443 (Fig. 6). The s-face of *D*_4_(2) is the face 1_2_. Therefore, *p*_2_(3_1_) = *p*_2_(1_2_) = 3.
  - We construct *D*_3_(3_1_) & *D*_4_(2) by gluing *D*_3_(3_1_) to *D*_4_(2) (Fig. 7). Since the k-peak is contributed by three polyhedra (polyhedra 1 and 2, and *D*_3_(3_2_)), the face 3_2_, should be glued to the face *ID*_c−face_(3_1_) (face *abfe*). Thus, *p*_2_(3_2_) = *p*_2_(*ID*_c−face_(3_1_)) = *p*_2_(2_2_) = 4.
  - We construct *D*_3_(3_2_) from the partial *p*_3_-codeword 34, glue it to *D*_4_(2), and obtain 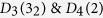 (Fig. 8). Since the k-peak is contributed by two polyhedra (polyhedron 1 and *D*_3_(3_2_)), the face 3_3_ will create a new ridge. Thus, *p*_2_(3_3_) = *r*(*N*_ridge_(3_2_) + 1) = *r*(9) = 4.
  - For the same reason, *p*_2_(3_4_) = *r*(10) = 4, and *p*_2_(3_5_) = *r*(11) = 3.
  - When we decode *p*_2_(3_1_)*p*_2_(3_2_)*p*_2_(3_3_)*p*_2_(3_4_)*p*_2_(3_5_) = 34443, a polyhedron is completed, thereby it turns out *p*_3_(3) = 34443.
4. In a similar way, *p*_3_(4) is determined to be *p*_2_(1_3_)*p*_2_(2_4_)*r*(12)*p*_2_(3_3_)*r*(13) = 34443. *p*_3_(5) = *p*_2_(1_4_)*p*_2_(2_3_)*p*_2_(3_4_)*p*_2_(4_3_)*r*(14) = 34443. *p*_3_(6) = *p*_2_(2_5_)*p*_2_(3_5_)*p*_2_(5_5_)*p*_2_(4_5_) = 3333.
5. When we decode *p*_3_(1)*p*_3_(2)*p*_3_(3)*p*_3_(4)*p*_3_(5)*p*_3_(6), a polychoron is completed, thereby it turns out *p*_4_[*A*] = 3333 34443 34443 34443 34443 3333.

### Generalization to higher dimensional polytopes

The *p*_4_-code can be generalized to the *p*_*n*_-code for *n*-polytopes (see Supplementary Note and Supplementary Table S1). The *p*_*n*_-codeword instructs how to construct the *n*-polytope from its building block (*n* − 1)-polytopes. However, as in the case of *p*_4_, *p*_*n*_ is redundant. By reducing the redundancy, we can obtain 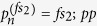. Here, 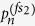 is the *n*-dimensional generalization of 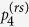. The superscript “*fs*_2_” indicates the 2-face-sequence codeword. The *i*-face is the *i*-dimensional face of an *n*-polytope. For example, a 2-face of a polychoron is a ridge, and a 1-face of a polychoron is a peak.

As an example, we explain *p*_*n*_s for *n*-dimensional cubes (*n*-cubes), and then demonstrate how the *p*_*n*_s are converted into their corresponding 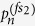
s. The 3-cube is an ordinary cube, and *p*_3_[3-cube] = 444444 = 4^6^. The 4-cube is composed of eight 3-cubes, and *p*_4_[4-cube] = 4^6^ 4^6^ 4^6^ 4^6^ 4^6^ 4^6^ 4^6^ 4^6^ = *p*_3_[3-cube]^8^. The 5-cube is composed of ten 4-cubes, and 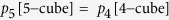
10. In general, an *n*-cube consists of 2*n (n* − 1) − cubes^20^, and *p*_*n*_[*n*-cube] = *p*_*n*−1_[(*n* − 1) − cube]^2*n*^ (for *n* ≥ 3).

The number of 1-faces of each 2-face of an *n*-polytope is (*n* − 2)! times recorded in *p*_*n*_, so that reducing the redundancy has a greater impact for higher dimensional polytopes. The redundancy can be reduced by using *fs*_2_ (see Supplementary Note and Supplementary Figure S1), which is denoted as


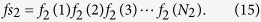


Here, *f*_2_(*i*) is the number of 1-faces of the 2-face *i. N*_2_ is the number of 2-faces of the *n*-polytope. For example, *p*_*n*_(*n*-cube) can be recovered from 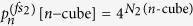. Here, *N*_2_(*n* - cube) is the number of 2-faces of the *n*-cube: *N*_2_(*n*-cube) = *n*(*n* − 1)2^*n*−3^.

Moreover, we can rewrite 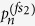 as *p*. In other words, we unify 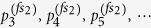 into *p*. Although the subscript “*n*” is removed, the dimension *n* of the polytope can be determined as a result of decoding *p*. We stress that polytopes of different dimensions can be represented by codewords of the same format, namely two number sequences separated by “;”.

## Discussion

E. A. Lazar, *et al*. introduced the Weinberg code to describe single Voronoi polyhedra^11^. But the Weingberg code does not allow for describing complexes of Voronoi polyhedra. On the other hand, our *p*-code allows us to describe complexes of Voronoi polyhedra, which would reveal the longer-range order of amorphous materials that cannot be seen from single Voronoi polyhedra. Our methods can be used to study a wide range of systems which are represented by polytopal tilings such as atoms in materials, grains in crystals, foams, galaxies in the universe, hyperspheres in higher-dimensional spaces, etc.^1^,^2^,^3^,^4^,^5^,^6^,^7^,^8^,^9^,^10^,^11^,^12^,^21^.

## Conclusion

We have developed a unified theory for representing polyhedral tilings and polytopes of different dimensions by brief codewords. Specifically, we have first formulated a method to deduce how to assemble ridges to form a polyhedral tiling or a polychoron from *rs* = *r*(1)*r*(2)*r*(3) 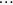 *r*(*R*). This has been achieved by reducing the redundancy in *p*_4_. Many polychora can be constructed just from *rs*, but there are some polychora that need *pp* which contains additional information about how to assemble ridges. It is remarkable that a mere sequence of *r*(*i*)s contains all or almost all information about how to assemble *r*(*i*)-gonal ridges to form a polychoron. Since a polychoron can be constructed from 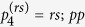, the polychoron can be represented by 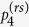. The local tiling structure composed of a central polyhedron and polyhedra surrounding the central polyhedron can also be represented by 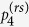, for it can be regarded as a part of a polychoron. Therefore, a polyhedral tiling can be characterized by distribution of 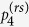
s of different central polyhedra. The idea of assembling two-dimensional components has been generalized to higher dimensional polytopes. Using the present method, *p*_*n*_ of an *n*-polytope can be converted into *p* whose length is as long as 1/(*n* − 2)! times of that of *p*_*n*_. Therefore, the impact of the present method factorially increases as the dimension of a polytope increases. The amount of tasks needed to convert *p*_*n*_ to *p* is negligible compared to that needed to generate *p*_*n*_. We stress that no subscript “*n*” that indicates the dimension of a polytope is attached to *p*. The dimension of the polytope is determined as a result of decoding *p*. In other words, the *p*_3_-code, *p*_4_-code, *p*_5_-code, 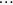, and *p*_*n*_-code have been unified into the *p*-code. Since shorter codewords are easier to handle for both humans and computers, our unified theory of polytopes would be a powerful tool to study a wide range of structures such as atoms in materials, grains in crystals, foams, galaxies in the universe, hyperspheres in higher-dimensional spaces, etc^1^,^2^,^3^,^4^,^5^,^6^,^7^,^8^,^9^,^10^,^11^,^12^,^21^.

## Additional Information

**How to cite this article**: Nishio, K. and Miyazaki, T. Describing polyhedral tilings and higher dimensional polytopes by sequence of their two-dimensional components. *Sci. Rep.* **7**, 40269; doi: 10.1038/srep40269 (2017).

**Publisher's note:** Springer Nature remains neutral with regard to jurisdictional claims in published maps and institutional affiliations.

## Supplementary Material

Supplementary Information
