# Supplementary material for: Describing polyhedral tilings and higher dimensional polytopes by sequence of their two-dimensional components
Source: Sci Rep. 2017 Jan 17;7:40269. doi: 10.1038/srep40269 (PMC5240342; doi:10.1038/srep40269)
Supplement: Supplementary Information [file srep40269-s1.pdf]

Supplemental Information for "Describing polyhedral tilings and higher dimensional polytopes by sequence of their two-dimensional components"

Kengo Nishio and Takehide Miyazaki

Correspondence should be addressed to K. N. (k-nishio@aist.go.jp).

Contents

Supplementary Table S1
Supplementary Figure S1
Supplementary Note
1. How to generate $p_3 = ps_2; sp$.
2. How to generate $p_4 = ps_3; sp$.
3. How to recover $p_4[A]$ from $p_4^*[A]$.
4. Code for n-polytopes.
5. How to construct $p_n^{(fs_2)}$ and recover p_n from $p_n^{(fs_2)}$.
6. Unify $p_n^{(fs_2)}$-representations into p-representation.

n-polytope		
$(n-1)$-face / block	block	
$(n-2)$-face / panel	$(n-2)$-bface / bpanel / subblock	subblock
$(n-3)$-face / joint	$(n-3)$-bface / bjoint	$(n-3)$-sface / sjoint

polychoron		
3-face / cell / polyhedron	polyhedron	
2-face / ridge	face / polygon	polygon
1-face / peak	edge	side

Supplementary Table S1 | Parts of n-polytope, blocks, and subblocks.

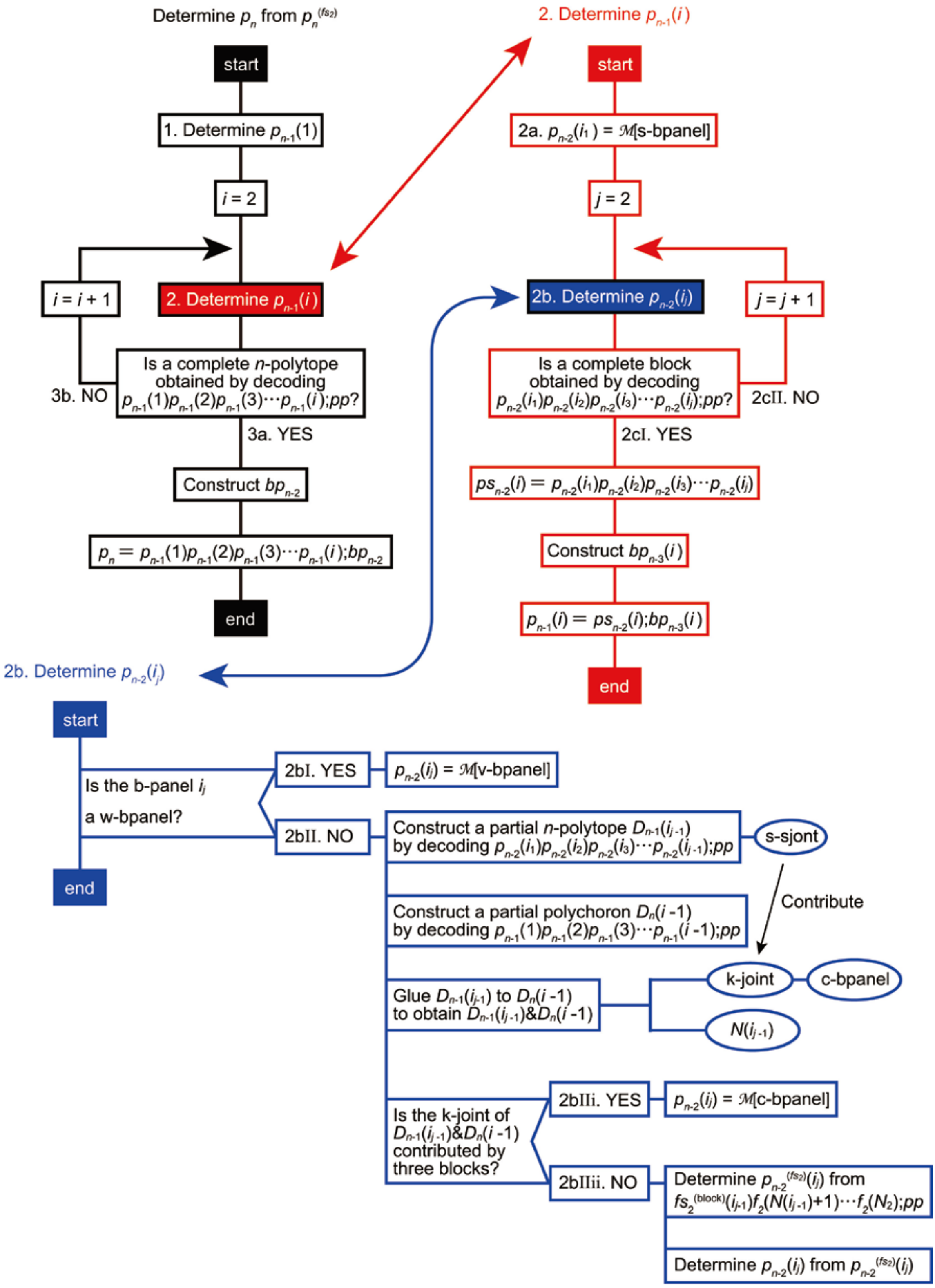

Supplementary Figure S1 | Procedures for recovering p_n from $p_n^{(fs_2)} = fs_2; pp$.

Supplemental Note

1. How to generate $\boldsymbol{p_3 = ps_2; sp}$. A polychoron associated with a disordered structure is simple, and a simple polychoron is composed of simple polyhedra. We explain how to generate the p_3-codeword of a simple polyhedron[12]. Note that a simple polychoron is the one whose 0-faces are all degree four; the 0-face is a point on the polychoron, where the vertices of polyhedra meet; the degree of a 0-face is the number of peaks incident to the 0-face. A simple polyhedron is the one whose vertices are all degree three; the degree of a vertex is the number of edges incident to the vertex.

p_3 represents a polyhedron, and consists of ps_2 and sp. We first describe how to generate ps_2. ps_2 is denoted as

$$ps_2 = p_2(1)p_2(2)p_2(3)\cdots p_2(F). \quad (1)$$

Here, $p_2(i)$ is the number of sides on the polygon i, and F is the number of polygons on the polyhedron. Generating ps_2 reduces to assigning IDs to polygons. To visually distinguish already-encoded polygons from to-be-encoded ones, we assume that all polygons are coloured at first, and make each polygon transparent when encoded. In encoding, we call a side of a transparent polygon glued to a coloured one a dangling side. To identify each side, we call the side j of the polygon i the side i_j. The side-ID i_j represents an integer: $i_j = j + \sum_{k=1}^{i-1} p_2(k)$. We abbreviate the smallest-ID dangling side as the s-side. The ps_2-codeword is generated as follows:

1.
   - (a) Choose a polygon and a side of that polygon as the initial polygon and side, respectively.
   - (b) The initial polygon is the polygon 1.
   - (c) Assign IDs $(1_1, 1_2, 1_3, \cdots, 1_{p_2(1)})$ to the sides of the polygon 1 from the initial side in a clockwise (CW) direction.
   - (d) Make the polygon 1 transparent.

2.
   - (a) The next polygon i $(2 \le i \le F)$ is the coloured one glued to the s-side.
   - (b) Assign IDs $(i_1, i_2, i_3, \cdots, i_{p_2(i)})$ to the sides of the polygon i from the one glued to the s-side in a CW direction.
   - (c) Make the polygon i transparent.

3.

(a) Repeat the procedure 2 until all polygons get transparent.

We assign edge IDs as follows. Given that two sides contribute to an edge, we first tentatively assign the smaller side ID to the edge, and then relabel the IDs so that the edge i is the one with the ith smallest tentative ID.

To explain sp, we first introduce the *zeroth tentative side-pairing* codeword ($tsp^{(0)}$). For this purpose, we define a *plot* as follows. A plot consists of a single dangling side or a chain of dangling sides. Here, two dangling sides are considered to be *chained* when they contribute to the same vertex contributed by two transparent polygons. Let x be the smallest side ID of a plot. We define the ID of that plot as x. We call the smallest-ID plot the *s-plot*. Let $P_3(i)$ be the partially transparent polyhedron obtained when the polygon i gets transparent. On $P_3(i)$, the polygon i is glued to the s-plot of $P_3(i-1)$. If the polygon i is glued to plots other than the s-plot, we call them *additional plots* (*a-plots*). Suppose that a plot y is an a-plot and that the side y (of the a-plot y) is glued to the side x (of the polygon i). We call such a pair of sides a *side-a-pair* yx. By collecting the side-a-pairs, $tsp^{(0)}$ is denoted as

$$tsp^{(0)} = y_a(1)x_a(1)y_a(2)x_a(2)y_a(3)x_a(3) \cdots y_a(N_{\text{side-a-pair}})x_a(N_{\text{side-a-pair}}), \quad (2)$$

Here, $y_a(i) > x_a(i)$ and $y_a(i) < y_a(i+1)$. $N_{\text{side-a-pair}}$ is the number of side-a-pairs. By a side-a-pair $y_a(i)x_a(i)$, we mean that the sides $y_a(i)$ and $x_a(i)$ should be glued together.

Using the decoding algorithm explained in the main text, the original polyhedron is recovered from $ps_2; tsp^{(0)}$. However, $tsp^{(0)}$ is redundant. We reduce the redundancy in $tsp^{(0)}$ step-by-step. To examine $y_a(N_{\text{side-a-pair}})x_a(N_{\text{side-a-pair}})$ for necessity, we consider the first test codeword denoted as

$$test^{(1)} = y_a(1)x_a(1)y_a(2)x_a(2)\, y_a(3)x_a(3) \cdots y_a(N_{\text{side-a-pair}}-1)x_a(N_{\text{side-a-pair}}-1), \quad (3)$$

which is obtained by stripping $y_a(N_{\text{side-a-pair}})x_a(N_{\text{side-a-pair}})$ off from $tsp^{(0)}$. Then we attempt to decode $ps_2; test^{(1)}$. If the original polyhedron is successfully reproduced,

then we say the side-a-pair $y_a(N_{\text{side-a-pair}})x_a(N_{\text{side-a-pair}})$ to be *curable*. Otherwise, we call the side-a-pair the *non-curable side-a-pair* (*side-na-pair*). If the side-a-pair is curable, then we can remove $y_a(N_{\text{side-a-pair}})x_a(N_{\text{side-a-pair}})$ from $tsp^{(0)}$. We therefore set the first tentative side-pairing codeword as $tsp^{(1)} = test^{(1)}$. On the other hand, if the side-a-pair is non-curable, then $tsp^{(1)} = tsp^{(0)}$.

To examine $y_a(N_{\text{side-a-pair}} + 1 - i)x_a(N_{\text{side-a-pair}} + 1 - i)$ for curability ($2 \le i \le N_{\text{side-a-pair}}$), we consider the ith test codeword $test^{(i)}$, which is obtained by stripping $y_a(N_{\text{side-a-pair}} + 1 - i)x_a(N_{\text{side-a-pair}} + 1 - i)$ off from $tsp^{(i-1)}$. Then we attempt to decode $ps_2; test^{(i)}$. If the side-a-pair $y_a(N_{\text{side-a-pair}} + 1 - i)x_a(N_{\text{side-a-pair}} + 1 - i)$ is curable, then $tsp^{(i)} = test^{(i)}$, otherwise, $tsp^{(i)} = tsp^{(i-1)}$.

We repeat the above-mentioned procedure, and $tsp^{(N_{\text{side-a-pair}})}$ is what we call sp. The sp–codeword is denoted as

$$sp = y(1)x(1)y(2)x(2)y(3)x(3)\cdots y(N_{\text{s}} - 1)x(N_{\text{s}} - 1). \qquad (4)$$

Here, the pair of sides $y(i)$ and $x(i)$ form the ith *non-curable additional pair* $y(i)x(i)$ (side-na-pair $y(i)x(i)$ for short). $y(i) > x(i)$ and $y(i) < y(i+1)$. N_{s} is the number of side-na-pairs.

2. How to generate $p_4 = ps_3; sp$. The p_4-codeword is a generalization of p_3, and consists of ps_3 and fp[12]. We first describe how to generate ps_3. ps_3 is denoted as

$$ps_3 = p_3(1)p_3(2)p_3(3)\cdots p_3(C). \qquad (5)$$

Here, $p_3(i)$ is the p_3-codeword of the polyhedron i, and C is the number of polyhedra on the polychoron. Generating ps_3 reduces to assigning polyhedron IDs. We assume that all polyhedra are coloured at first, and make each polyhedron transparent when encoded. In encoding, we call a face of a transparent polyhedron glued to a coloured one a dangling face. To identify each face (edge), we call the face j (edge j) of the polygon i the face i_j (edge i_j). The face-ID i_j (edge-ID i_j) represents an integer: $i_j = j + \sum_{k=1}^{i-1} F(k)$ ($i_j = j + \sum_{k=1}^{i-1} E(k)$). Here, $F(k)$ ($E(k)$) is the number of faces (edges) on the polyhedron k. We abbreviate the smallest-ID dangling face as the *s-face*. The ps_3-codeword is generated as follows:

1.

(a) Choose a polyhedron, a face of that polyhedron, and an edge of that face as the initial polyhedron, face and edge, respectively

(b) The polyhedron 1 is the initial polyhedron.

(c) Determine $p_3(1)$ by encoding the polyhedron 1 in such a way that the face 1_1 (edge 1_1) becomes the initial face (edge).

(d) Make the polyhedron 1 transparent.

2.

(a) The next polyhedron i $(2 \leq i \leq C)$ is the coloured one glued to the s-face.

(b) Determine $p_3(i)$ by encoding the polyhedron i in such a way that the face i_1 (edge i_1) is glued to the s-face (the smallest-ID edge of the s-face).

(c) Make the polyhedron i transparent except for the vertices and edges.

3.

(a) Repeat the procedure 2 until all polyhedra get transparent.

We assign peak (ridge) IDs as follows. Given that three edges (two faces) contribute to a peak (ridge), we first tentatively assign the smallest edge (face) ID to the peak (ridge), and then relabel the IDs so that the peak (ridge) i is the one with the ith smallest tentative ID.

To define *zeroth tentative face-pairing codeword* ($tfp^{(0)}$), we explain plots for polychora. If a pair of two dangling faces contribute to a peak contributed by two transparent polyhedra, the dangling faces are considered to be *chained*. Chained dangling faces form a plot. A single dangling face also forms a plot. The plot here is a two-dimensional object. We assign plot IDs so that the smallest-ID face of the plot *x* is the face *x*. The polyhedron i in $P_4(i)$ is glued to the s-plot of $P_4(i-1)$. If the polyhedron i is glued to plots other than the s-plot, they are called a-plots. Here, $P_4(k)$ is the partially transparent polychoron obtained when the polyhedron k gets transparent. By the *face-a-pair* wzv, we mean that the face w (of the polyhedron i) is glued to the face v of the a-plot v in such a way that the edge contributed by the side z (of the polygon w) is glued to the smallest-ID edge of the face v. By collecting the face-a-pairs, $tfp^{(0)}$ is denoted as

$$tfp^{(0)} = w_a(1)z_a(1)v_a(1) \\ \cdots w_a(N_{\text{face-a-pair}})z_a(N_{\text{face-a-pair}})v_a(N_{\text{face-a-pair}}). \quad (6)$$

Here, $w_a(i) > v_a(i)$ and $w_a(i) < w_a(i+1)$. $N_{\text{face-a-pair}}$ is the number of face-a-pairs.

By a similar argument for sp, the redundancy in $tfp^{(0)}$ can be removed step-by-step with generating $tfp^{(1)}$, $tfp^{(2)}$, $tfp^{(3)}$, ⋯, and $tfp^{(N_{\text{face-a-pair}})}$ is what we call *fp*.

3. How to recover $p_4[A]$ from $p_4^*[A]$. We first decode $p_4^*[A] = 3333344433444334 443344433333$ using the p_3-code. When the 4th digit is decoded, a 3333-polyhedron is completed, thereby it turns out $p_3(1) = 3333$. We then remove 3333 from $p_4^*[A]$, and obtain $p_4^{*(-1)}[A] = 3444334443344433444333333$. To find out $p_3(2)$, we decode $p_4^{*(-1)}[A]$. When a 34443-polyhedron is obtained, it turns out $p_3(2) = 34443$. We then remove 34443 from $p_4^{*(-1)}[A]$, and obtain $p_4^{*(-2)}[A] = 34443344433444333333$. By repeating this procedure, $p_4[A]$ is determined to be 3333 34443 34443 34443 34443 3333.

4. Code for *n*-polytopes. We have formulated the p_3-code for polyhedra, and then generalized it to the p_4-code for polychora in the previous paper[12]. Its summary has been given above and in the main text. The p_3-code (p_4-code) consists of (1) an encoding algorithm for converting a polyhedron (polychoron) into $p_3 = ps_2; sp$ ($p_4 = ps_3; fp$), (2) an algorithm for assigning IDs to the parts of that polyhedron (polychoron), and (3) a decoding algorithm for recovering the original polyhedron (polychoron) from p_3 (p_4). Note that a polyhedron (polychoron) is a 3-polytope (4-polytope). Now we will recursively generalize the p_3-code and the p_4-code to the p_n-code and show that an n-polytope can be represented by a p_n-codeword, which is denoted by

$$p_n = ps_{n-1}; bp_{n-2}. \quad (7)$$

Here, ps_{n-1} is the $(n-1)$-*polytope-sequence* codeword, and bp_{n-2} is the *bpanel-pairing* codeword. Note that, for $n = 3$ and 4, the bp_{n-2}-codewords are $bp_1 = sp$ and $bp_2 = fp$, respectively.

To formulate p_n, we generalize terms used for p_3 and p_4. As we regard that a polyhedron is a tiling by polygons, we regard an n-polytope as a tiling by $(n-1)$-polytopes of the surface of an n-dimensional object that is topologically the same as an $(n-1)$-sphere. We call the building-block $(n-1)$-polytopes *blocks*. Note that, for

$n = 3$, blocks for a polyhedron are polygons. Hereafter, we assume $0 \leq m \leq n-2$. As we distinguish the vertices and edges of a polyhedron from the corners and sides of a polygon, respectively, we distinguish m-faces of an n-polytope from those of a block. We therefore call m-faces of a block *m-bfaces*. We give specific names to the parts that play a key role in formulating the p_n-code. We call the $(n-2)$-face of an n-polytope a *panel* and the $(n-3)$-face a *joint*. Similarly, we call the $(n-2)$-bface of a block a *bpanel* and the $(n-3)$-bface a *bjoint*. Note that, for $n = 3$, a panel and a joint of a polyhedron are an edge and a vertex, respectively. A bpanel and a bjoint of a polygon are a side and a corner, respectively. We assume that the blocks are glued together such that each bpanel of each block meets exactly one other block along a panel of the n-polytope. Since we consider simple polytopes, every joint is contributed by three blocks.

We also introduce *subblocks*. As an n-polytope is a tiling by blocks, each block is a tiling by $(n-2)$-polytopes. We call the building-block $(n-2)$-polytopes subblocks. Hereafter, we assume $1 \leq l \leq n-3$, and call l-faces of a subblock l-sfaces. In particular, we call the $(n-3)$-sface a *sjoint*. The relations between parts of an n-polytope, a block, and a subblock are summarized in Supplementary Table S1. The subblocks are glued together such that each sjoint of each subblock meets exactly one other subblock along a bjoint of the block. A bpanel of a block is a subblock. But when we call a subblock, we regard it as a building block of a block. For example, we say that each l-sface of a subblock contributes to an l-bface of a bpanel.

Using the terms introduced above, ps_{n-1} is denoted as

$$ps_{n-1} = p_{n-1}(1)p_{n-1}(2)p_{n-1}(3) \cdots p_{n-1}(N_{\text{block}}). \qquad (8)$$

Here, $p_{n-1}(i)$ is the p_{n-1}-codeword of the block i. N_{block} is the number of blocks forming the n-polytope.

Generating ps_{n-1} reduces to assigning IDs to blocks. All blocks are coloured at first, and we make each block transparent when encoded. In encoding, we call a bpanel of a transparent block glued to a coloured one a *dangling bpanel*. To identify each m-bface, we assign an ID i_j to it. By the m-bface i_j, we mean the m-bface j of the block i. Let $N_{m-\text{bface}}(x)$ be the number of m-bfaces on the block x. The m-bface-ID i_j represents an integer: $i_j = j + \sum_{x=1}^{i-1} N_{m-\text{bface}}(x)$. We abbreviate the smallest-ID dangling bpanel as the *s-bpanel*. We recursively define the *l-sbface* of a bpanel as follows: (1) for $l = n-3$, the l-sbface is the smallest-ID l-bface of that bpanel and (2) for $l < n-3$, the l-sbface is the smallest-ID l-bface of the $(l+1)$-sbface.

Using the p_{n-1}-code, we can convert a block into p_{n-1} and assign IDs to m-bfaces of that block. The ps_{n-1}-codeword of an n-polytope is generated using the p_{n-1}-code as follows:

1.
   (a) Choose a block as the initial block, an $(n-2)$-bface of the initial block as the initial $(n-2)$-bface, an $(n-3)$-bface of the initial $(n-2)$-bface as the initial $(n-3)$-bface, and so on.
   (b) Determine $p_{n-1}(1)$ by encoding the block 1 using the p_{n-1}-code in such a way that the bpanel 1_1 is the initial bpanel and each initial l-bface becomes the l-bface 1_1.
   (c) Make the block 1 transparent.

2.
   (a) The next block i $(2 \le i \le N_{\text{block}})$ is the coloured one glued to the s-bpanel.
   (b) Determine $p_{n-1}(i)$ by encoding the block i in such a way that the bpanel i_1 is glued to the s-bpanel and each l-bface i_1 is glued to the l-sbface of the s-bpanel.
   (c) Make the block i transparent.

3.
   (a) Repeat the procedure 2 until all blocks get transparent.

We can assign an ID to each m-face of the n-polytope as follows. Given that $(n-m)$ m-bfaces contribute to an m-face, we first tentatively assign the smallest m-bface ID to the m-face, and then relabel the IDs so that the m-face i is the one with the ith smallest tentative ID.

To formulate bp_{n-2} of p_n, we first define the *zeroth tentative bpanel-pairing codeword* ($tbp_{n-2}^{(0)}$). For this purpose, we explain plots for an n-polytope. If a pair of two dangling bpanels contribute to a joint contributed by two transparent blocks, the dangling bpanels are considered to be *chained*. Chained dangling bpanels form a plot. A single dangling bpanel forms a plot as well. The plot here is an $(n-2)$-dimensional object. We assign plot IDs so that the smallest-ID bpanel of the plot *x* is the bpanel *x*. The block i of $P_n(i)$ is glued to the s-plot of $P_n(i-1)$. Here, $P_n(i)$ is the partially transparent n-polytope obtained when the block i gets transparent. If the block i is glued to plots other than the s-plot, they are called a-plots. By the *bpanel-a-pair*

$w_a s_a^{n-3} s_a^{n-4} \cdots s_a^2 s_a^1 v_a$, we mean that the bpanel w_a (of the block i) is glued to the bpanel v_a (of the a-plot v_a of $P_n(i-1)$) in such a way that each l-bface (of the bpanel w_a) contributed by the l-sface s_a^l (of the subblock w_a) is glued to the smallest-ID l-bface of the bpanel v_a. By collecting the bpanel-a-pairs, $tbp_{n-2}^{(0)}$ is obtained as

$$tbp_{n-2}^{(0)} = a(1)a(2)a(3) \cdots a\left(N_{\text{bpanel-a-pair}}\right), \qquad (9)$$

Here, $a(i) = w_a(i) s_a^{n-3}(i) s_a^{n-4}(i) \cdots s_a^2(i) s_a^1(i) v_a(i)$. $w_a(i) > v_a(i)$ and $w_a(i) < w_a(i+1)$. $N_{\text{bpanel-a-pair}}$ is the number of bpanel-a-pairs on the n-polytope.

To formulate the decoding algorithm for recovering the original n-polytope from $ps_{n-1}; tbp_{n-2}^{(0)}$, we introduce some definitions. In decoding, if a bpanel of a block of the partial n-polytope is not glued to the other bpanel, we call it a dangling bpanel. We also call the smallest-ID dangling bpanel the s-bpanel. We regard that a bundle of m-bfaces meeting along an m-dimensional region forms an m-face of the partial n-polytope. An isolated m-bface also forms an m-face. In a partial n-polytope, if the pair of dangling bpanels contribute to a joint that is also contributed by three blocks, we call that joint an *illegal joint* (*i-joint*). When an i-joint is generated, we rectify it by gluing together the two dangling bpanels contributing to it.

Using the p_{n-1}-code, we can recover the original $(n-1)$-polytope from p_{n-1}. The n-polytope can be recovered from $ps_{n-1}; tpp_{n-2}^{(0)}$ using the p_{n-1}-code as follows:

1.
   (a) Decode $p_{n-1}(1)$ using the p_{n-1}-code to obtain the block 1, assigning bpanel IDs and l-bface IDs.

2.
   (a) Decode $p_{n-1}(i)$ to obtain the next block i $(2 \le i \le N_{\text{block}})$, assigning bpanel IDs and l-bface IDs.
   (b) Glue the bpanel i_1 to the s-bpanel of the partial n-polytope in such a way that each l-bface i_1 is glued to the l-sbface of the s-bpanel.
   (c) If $w(x)$ $\left(1 \le x \le N_{\text{bpanel-a-pair}}\right)$ is the bpanel ID of the block i, then glue the bpanel $w(x)$ to the bpanel $v(x)$ of the partial n-polytope in such a way that each l-bface (of the bpanel $w(x)$) contributed by the l-sface $s_a^l(x)$ (of the subblock $w(x)$) is glued to the smallest-ID l-bface of the bpanel $v(x)$.

(d) If i-joints are generated, then rectify them, and repeat this procedure until no i-joints remain.

3.

(a) Repeat the procedure 2 until all blocks are placed.

By a similar argument for sp and fp, the redundancy in $tbp_{n-2}^{(0)}$ can be removed step-by-step with generating $tbp_{n-2}^{(1)}$, $tbp_{n-2}^{(2)}$, $tbp_{n-2}^{(3)}$, ⋯, and $tbp_{n-2}^{(N_{\text{bpanel-a-pair}})}$ is what we call bp_{n-2}. The bp_{n-2}-codeword is denoted as,

$$bp_{n-2} = na(1)na(2)na(3) \cdots na(N_{\text{b}}). \quad (10)$$

Here, $na(i) = w(i)s^{n-3}(i)s^{n-2}(i) \cdots s^2(i)s^1(i)v(i)$. By the *bpanel-na-pair* $na(i)$, we mean that the bpanels $w(i)$ and $v(i)$ should be glued together in such a way that each l-bface contributed by the l-sface $s^l(i)$ (of the subblock $w(i)$) is glued to the smallest-ID l-bface of the bpanel $v(i)$. $w(i) > v(i)$ and $w(i) < w(i+1)$. N_{b} is the number of bpanel-na-pairs.

5. How to construct $p_n^{(fs_2)}$ and recover p_n from $p_n^{(fs_2)}$. By reducing the redundancy in p_4, we have formulated the $p_4^{(rs)}$-code for polychora (4-polytopes) in the main text. Using the $p_4^{(rs)}$-code, a polychoron is represented by $p_4^{(rs)} = rs; pp$. Now we will recursively generalize the $p_4^{(rs)}$-code to the $p_n^{(fs_2)}$-code and show that an n-polytope can be represented by a $p_n^{(fs_2)}$-codeword, which is denoted as

$$p_n^{(fs_2)} = fs_2; pp. \quad (11)$$

Here, fs_2 is the 2-face-sequence codeword, which is denoted as

$$fs_2 = f_2(1)f_2(2)f_2(3) \cdots f_2(N_2), \quad (12)$$

$f_2(i)$ is the number of 1-faces on the 2-face i, N_2 is the number of 2-faces on the n-polytope. Note that a face and a ridge are the 2-faces of a polyhedron and a polychoron, respectively, and that the $p_3^{(fs_2)}$- and $p_4^{(fs_2)}$-codes are identical with the p_3- and $p_4^{(rs)}$-codes, respectively.

For $p_4^{(rs)}$, we have unified sps (written in local side ID) and fp (written in global face ID) into pp (written in part ID) as follows:

1. We have translated $sp(i)$ into global side ID, and obtained $sp^g(i)$.
2. We have put together $sp^g(i)$s into $sp^{g*} = sp^g(1)sp^g(2)sp^g(3)\cdots sp^g(C)$.
3. We have put together sp^{g*} and fp into $sp^{g*};fp$.
4. We have translated $sp^{g*};fp$ into part ID, and then removed the separator ";". As a result, we have obtained pp of $p_4^{(rs)}$.

Similarly, we construct pp of $p_n^{(fs_2)}$ as follows:

1. We translate $bp_l(i)$ of $p_{l+2}(i)$ into global x-face ID ($1 \leq x \leq l$), and obtain $bp_l^g(i)$.
2. We put together $bp_l(i)$s into $bp_l^{g*} = bp_l^g(1)bp_l^g(2)\cdots bp_l^g\left(N_{(l+2)-\text{polytope}}\right)$, where $N_{(l+2)-\text{polytope}}$ is the number of $(l+2)$-polytopes on the n-polytope.
3. We put together bp_1^{g*}, bp_2^{g*},⋯, bp_{n-2}^{g*} into $bp_1^{g*};bp_2^{g*};\cdots;bp_{n-2}^{g*}$.
4. We translate $bp_1^{g*};bp_2^{g*};\cdots;bp_{n-2}^{g*}$ into part ID, and then remove the separators ";". As a result, we obtain pp of $p_n^{(fs_2)}$.

For the procedure 4, we assign a unique ID to each part, which we call a part ID. For this purpose, we define the part set. In the main text, we have defined the set of parts of the polygon i as $S[\text{polygon } i] = \left\{\text{polygon } i, \text{side } i_1, \cdots, \text{side } i_{p_2(i)}\right\}$. To define the part set of higher-dimensional polytopes, we introduce some notations. We write $\langle k, i\rangle$ for the k-polytope i. For example, $\langle 2, i\rangle$ is the polygon i. $\langle \alpha, j, k, i\rangle$ denotes the α-face j of the k-polytope i. For example, $\langle 1, j, 2, i\rangle$ is the side j of the polygon i, in other words, the side i_j. $N(\alpha, k, i)$ is the number of α-faces on the k-polytope i. For example, $N(1,2,i)$ is the number of sides on the polygon i. With these notations, $S[\text{polygon } i]$ can be rewritten as

$$S[\text{polygon } i] = S[\langle 2, i\rangle] = \{\langle 2, i\rangle, \langle 1,1,2,i\rangle, \cdots, \langle 1, N(1,2,i), 2, i\rangle\}. \quad (13)$$

For $k > 2$, we recursively define $S[\langle k, i\rangle]$ as follows:

- For $i = 1$,
$$S[\langle k, 1\rangle] = \{S[\langle k-1,1\rangle], \langle k, 1\rangle, S[\langle k-1,2\rangle], \cdots, S[\langle k-1, N(k-1,k,1)\rangle],$$
$$\langle k-2,1,k,1\rangle, \cdots, \langle k-2, N(k-2,k,1), k, 1\rangle,$$

$$\langle k-3,1,k,1\rangle,\cdots,\langle k-3,N(k-3,k,1),k,1\rangle,$$
$$\vdots$$
$$\langle 1,1,k,1\rangle,\cdots,\langle 1,N(1,k,1),k,1\rangle\}$$
$$=\{S[\langle k-1,1\rangle],\langle k,1\rangle,S[\langle k-1,2\rangle],\cdots,S[\langle k-1,N(k-1,k,1)\rangle],$$
$$\langle k-2,1,k,1\rangle,\cdots,\langle 1,N(1,k,1),k,1\rangle\}. \quad (14)$$

- For $i \neq 1$,

$$S[\langle k,i\rangle]=\{\langle k,i\rangle,S[\langle k-1,i_1\rangle],\cdots,S[\langle k-1,i_{N(k-1,k,i)}\rangle],$$
$$\langle k-2,1,k,i\rangle,\cdots,\langle 1,N(1,k,i),k,i\rangle\}. \quad (15)$$

Note that the $S[\langle k,1\rangle]$ and $S[\langle k,i\rangle]$ ($i \neq 1$) are separately defined. This is because we will remove the subscript "n" and superscript "(fs_2)" from $p_n^{(fs_2)}$ to construct p later. Before decoding p, we do not know the dimension n of a polytope. The dimension n is determined as a result of decoding. In decoding, when $\langle k-1,1\rangle$ is completed and all the 2-faces are not placed, we know that the dimension of the polytope is k or more. Therefore, $\langle k,1\rangle$ follows $S[\langle k-1,1\rangle]$, and $S[\langle k-1,2\rangle]$ follows $\langle k,1\rangle$.

In particular, the set of parts of an n-polytope is defined as

$$S[\langle n,1\rangle]=\{S[\langle n-1,1\rangle],\langle n,1\rangle,S[\langle n-1,2\rangle],\cdots,S[\langle n-1,N(n-1,n,1)\rangle],$$
$$\langle n-2,1,n,1\rangle,\cdots,\langle 1,N(1,n,1),n,1\rangle. \quad (16)$$

Note that $\langle n,1\rangle$ is the n-polytope itself. We assign IDs to parts of the n-polytope in such a way that (1) IDs of $S[\langle 2,i\rangle]$ are assigned in the order of $\langle 2,i\rangle$, $\langle 1,1,2,i\rangle,\cdots$, $\langle 1,N(1,2,i),2,i\rangle$, (2) for $2<k$, IDs of $S[\langle k,1\rangle]$ are assigned in the order of $S[\langle k-1,1\rangle]$, $\langle k,1\rangle$, $S[\langle k-1,2\rangle],\cdots$, $S[\langle k-1,N(k-1,k,1)\rangle]$, $\langle k-2,1,k,1\rangle,\cdots$, $\langle 1,N(1,k,1),k,1\rangle$, and (3) for $2<k$ and $i \neq 1$, IDs of $S[\langle k,i\rangle]$ are assigned in the order of $\langle k,i\rangle$, $S[\langle k-1,i_1\rangle]$, $\cdots$, $S[\langle k-1,i_{N(k-1,k,i)}\rangle]$, $\langle k-2,i_1,k,i\rangle$, $\cdots$, $\langle 1,i_{N(1,k,1)},k,i\rangle$.

The bpanel-na-pairs of a k-polytope is identified from pp as follows. Let $p(i)$ be the ith digit of pp. If the part $p(i)$ is a $(k-2)$-face of a $(k-1)$-polytope forming a k-polytope and the part $p(i+k-2)$ is a $(k-2)$-face of a $(k-1)$-polytope forming the same k-polytope, then the pair $p(i)\cdots p(i+k-2)$ is a bpanel-na-pair of that k-polytope. We call the part $p(i)$ a *w-bpanel*, and the part $p(i+k-2)$ a *v-bpanel*.

When determining p_4 from $p_4^{(rs)}$, we have step-by-step determined $p_3(1),p_3(2),p_3(3),\cdots,p_3(C)$. To determine $p_3(i)$, we have step-by-step deduced

$p_2(i_1), p_2(i_2), p_2(i_3), \cdots, p_2(i_{F(i)})$. To deduce $p_2(i_j)$, we have examined whether the face i_j should be glued to an existing face of the partial polychoron or create a new ridge. Similarly, as is summarized in Supplementary Figure S1, to determine p_n from $p_n^{(fs_2)}$, we step-by-step determine p_{n-1}s of blocks. To determine $p_{n-1}(i)$ (p_{n-1} of the block i), we step-by-step deduce $p_{n-2}(i_j)$ (p_{n-2} of the subblock j of the block i). To deduce $p_{n-2}(i_j)$, we examine whether the bpanel i_j should be glued to an existing bpanel of the partial n-polytope or create a new panel.

We start with determining $p_{n-1}(1)$. Note that p_3 is identical with $p_3^{(fs_2)}$, but p_n is different from $p_n^{(fs_2)}$ for $n > 3$. Therefore, a two-step process is necessary to determine $p_{n-1}(1)$ for $n > 3$. In fact, we first determine $p_{n-1}^{(fs_2)}(1)$, and then recover $p_{n-1}(1)$ from $p_{n-1}^{(fs_2)}(1)$ using the $p_{n-1}^{(fs_2)}$-code. Here, $p_{n-1}^{(fs_2)}(i)$ is $p_{n-1}^{(fs_2)}$ of the block i. To distinguish fs_2 of an n-polytope and that of a block, we write $fs_2^{(\text{block})}(i)$ for fs_2 of the block i. Let X be the number of 2-faces on the block 1. By construction, the first X digits of fs_2 of $p_n^{(fs_2)}$ forms $fs_2^{(\text{block})}(1)$. However, we do not know X beforehand. To find out X, we decode $f_2(1)f_2(2)f_2(3)\cdots f_2(N_2); pp$ using the $p_{n-1}^{(fs_2)}$-code. When an $(n-1)$-polytope is completed, we count the number of 2-faces on the polytope. Suppose that there are α 2-faces on the polytope. Then $X = \alpha$, and $fs_2^{(\text{block})}(1) = f_2(1)f_2(2)f_2(3)\cdots f_2(\alpha)$. If bpanel-na-pairs are found in decoding, record them in $pp(1)$. By putting $fs_2^{(\text{block})}(1)$ and $pp(1)$ together, $p_{n-1}^{(fs_2)}(1) = fs_2^{(\text{block})}(1); pp(1)$. We determine $p_{n-1}(1)$ from $p_{n-1}^{(fs_2)}(1)$ by using the $p_{n-1}^{(fs_2)}$-code.

For $2 \leq i$, p_{n-1} of the block i can be determined from $p_n^{(fs_2)}$ and the partial ps_{n-1}-codeword $p_{n-1}(1)p_{n-1}(2)p_{n-1}(3)\cdots p_{n-1}(i-1)$. The p_{n-1}-codeword of the block i is denoted by

$$p_{n-1}(i) = p_{n-2}(i_1)p_{n-2}(i_2)p_{n-2}(i_3)\cdots p_{n-2}(i_{N_{\text{subblock}}(i)}); bp_{n-3}(i). \quad (17)$$

Here, $N_{\text{subblock}}(i)$ is the number of subblocks on the block i. Our first task is to deduce $p_{n-2}(i_1)$. For this purpose, we construct a partial n-polytope $D_n(i-1)$ by decoding $p_{n-1}(1)p_{n-1}(2)p_{n-1}(3)\cdots p_{n-1}(i-1); pp$. Since the bpanel i_1 (the bpanel 1 of the block i) is glued to the s-bpanel of $D_n(i-1)$, the bpanel i_1 should be the mirror image of that s-bpanel. Note that, when formulating the $p_4^{(rs)}$-code, there was no need to care about the mirror-image relation, for the mirror image of a k-gon is a k-gon. To determine $p_{n-2}(i_1)$, we encode the mirror image of the s-bpanel using the p_{n-2}-code. Now we need to carefully choose each initial l-face for encoding, so that the bpanel i_1 is glued to the s-bpanel in such a way that each l-bface i_1 of the bpanel i_1 is glued to

the l-sbface of the s-bpanel. To meet this requirement, $p_{n-2}(i_1)$ is determined as follows:

1. To properly choose initial faces, mark each l-sbface of the s-bpanel.
2. Construct the mirror image of the marked s-bpanel.
3. Encode the mirror-image $(n-2)$-polytope by choosing the marked l-faces as the initial ones.
4. The p_{n-2}-codeword thus obtained is $p_{n-2}(i_1)$.
5. We denote the relation between $p_{n-2}(i_1)$ and the s-bpanel as $p_{n-2}(i_1) = \mathcal{M}[\mathrm{s-bpanel}]$.

For $2 \le j$, $p_{n-2}(i_j)$ can be determined from $p_n^{(fs_2)}$, the partial ps_{n-1}-codeword $p_{n-1}(1)p_{n-1}(2)p_{n-1}(3)\cdots p_{n-1}(i-1)$, and the partial ps_{n-2}-codeword $p_{n-2}(i_1)$ $p_{n-2}(i_2)p_{n-2}(i_3)\cdots p_{n-2}(i_{j-1})$. To deduce $p_{n-2}(i_j)$, we examine whether the bpanel i_j should be glued to an existing bpanel of $D_n(i-1)$ or create a new panel. For this purpose, we construct the partial n-polytope $D_{n-1}(i_{j-1})\&D_n(i-1)$ as follows. We first construct the partial block $D_{n-1}(i_{j-1})$, which is obtained by decoding $p_{n-2}(i_1)$ $p_{n-2}(i_2)p_{n-2}(i_3)\cdots p_{n-2}(i_{j-1});pp$ using the p_{n-1}-code. We then glue the bpanel i_1 (of $D_{n-1}(i_{j-1})$) to the s-bpanel of $D_n(i-1)$ in such a way that each l-bface i_1 is glued to the l-sbface of the s-bpanel. Next we examine pp. If there is k such that the part $p(k)$ is a bpanel of $D_{n-1}(i_{j-1})$ and the part $p(k+n-2)$ is a bpanel of $D_n(i-1)$, then the part $p(k)$ is a w-bpanel and the part $p(k+n-2)$ is a v-bpanel. Therefore, we glue the w-bpanel and the v-bpanel together in such a way that each l-bface of the w-bpanel contributed by the part $p(k+l)$ is glued to the smallest-ID l-bface of the v-bpanel. When i-joints are generated, we rectify them until no i-joints remain. The partial n-polytope thus obtained is $D_{n-1}(i_{j-1})\&D_n(i-1)$.

To examine whether the bpanel i_j should be glued to an existing bpanel of $D_n(i-1)$ or create a new panel, we first examine whether the bpanel i_j is a w-bpanel that should be glued to an existing v-bpanel or not. The bpanel-na-pairs are recorded in pp using part IDs. The part ID of the bpanel i_j, or subblock i_j, is the number of parts of $D_{n-1}(i_{j-1})\&D_n(i-1)$ plus one. If there is k such that the $p(k)$ is the part ID of the bpanel i_j and the part $p(k+n-2)$ is a bpanel of $D_n(i-1)$, then the bpanel i_j is a w-bpanel and the part $p(k+n-2)$ is a v-bpanel. Therefore, the bpanel i_j should be glued to the v-bpanel in such a way that each l-bface contributed by the part $p(k+l)$ (of the subblock i_j) is glued to the smallest-ID l-bface of the v-bpanel. To meet this

requirement, the bpanel i_j should be the mirror image of the v-bpanel and $p_{n-2}(i_j)$ is determined by encoding the mirror image as follows:

1. Inscribe a number "$p(k+l)$" onto the smallest-ID l-bface of the v-bpanel.
2. Construct the mirror image of the inscribed v-bpanel.
3. Encode the mirror-image $(n-2)$-polytope so that the part IDs of the inscribed l-sfaces agree with the inscribed numbers.
4. The p_{n-2}-codeword thus obtained is $p_{n-2}(i_j)$.
5. We denote the relation between $p_{n-2}(i_j)$ and the v-bpanel as $p_{n-2}(i_j) = \mathcal{M}[\mathrm{v-bpanel}]$.

Even if the bpanel i_j is not a w-bpanel, there remains a possibility that it is glued to an existing bpanel. Specifically, when the bpanel i_j contributes to an i-joint, it will be glued to an existing bpanel to rectify the i-joint. To examine this possibility, we introduce some terms. In $D_{n-1}(i_{j-1})$, a sjoint of a subblock that is not glued to the other subblock is called a dangling sjoint. We also call the smallest-ID dangling sjoint *s-sjoint*. We refer to the joint contributed by the s-sjoint as the *key joint* (*k-joint*), for it plays a key role in determining whether the bpanel i_j should be glued to an existing bpanel or create a new panel. We write $ID_{\mathrm{k-joint}}(i_{j-1})$ for the global joint ID of the k-joint. There exists one dangling bpanel contributing to the k-joint, which we call a *candidate bpanel* (*c-bpanel*). We write $ID_{\mathrm{c-bpanel}}(i_{j-1})$ for the global bpanel ID of the c-bpanel. The bpanel i_j will be glued to the bpanel $ID_{\mathrm{c-bpanel}}(i_{j-1})$ or create a new panel. Now, we need to consider two cases:

(Case 1) The k-joint of $D_{n-1}(i_{j-1})\&D_n(i-1)$ is contributed by three blocks ($D_{n-1}(i_{j-1})$ and two from $D_n(i-1)$). In this case, in constructing $D_{n-1}(i_j)\&D_n(i-1)$, when the joint $ID_{\mathrm{k-joint}}(i_{j-1})$ is contributed by three blocks and the bpanel i_j is not glued to the bpanel $ID_{\mathrm{c-bpanel}}(i_{j-1})$, the joint will be illegal. To rectify it, the bpanels i_j and $ID_{\mathrm{c-bpanel}}(i_{j-1})$ should be glued together. Therefore, the bpanel i_j should be the mirror image of the c-bpanel, and $p_{n-2}(i_j)$ is determined by encoding the mirror image of the c-bpanel. Now we need to carefully choose each initial l-face for encoding so that the sjoint 1 of the subblock i_j should be glued to the s-sjoint. To properly choose each initial l-face, we hereafter assume $1 \le \mu \le n-4$, and recursively define the μ-*ssface* of a sjoint as follows: (1) for $\mu =$

$n-4$, the μ-ssface is the smallest-ID μ-sface of that sjoint and (2) for $\mu < n-4$, the μ-ssface is the smallest-ID μ-sface of the $(\mu+1)$-ssface. $p_{n-2}(i_j)$ is determined as follows:

1. Mark the bjoint of the c-bpanel that contributes to the k-joint and each μ-bface of the c-bpanel that contributes to the μ-face of the k-joint that is contributed by the μ-ssface of the s-sjoint.
2. Construct the mirror image of the marked c-bpanel.
3. Encode the mirror-image $(n-2)$-polytope by choosing the marked faces as the initial ones.
4. The p_{n-2}-codeword thus obtained is $p_{n-2}(i_j)$.
5. We denote the relation between $p_{n-2}(i_j)$ and the c-bpanel as $p_{n-2}(i_j) = \mathcal{M}[\mathrm{c-bpanel}]$.

(Case 2) The k-joint of $D_{n-1}(i_{j-1})\&D_n(i-1)$ is contributed by two blocks ($D_{n-1}(i_{j-1})$ and one from $D_n(i-1)$). In this case, the bpanel i_j should create a new panel. To determine $p_{n-2}(i_j)$, we determine $p_{n-1}^{(fs_2)}(i_j)$, which is the partial $p_{n-1}^{(fs_2)}$-codeword for $D_{n-1}(i_j)$. We write $fs_2^{(\mathrm{block})}(i_j)$ for fs_2 of $p_{n-1}^{(fs_2)}(i_j)$. The 2-faces existing in $D_{n-1}(i_{j-1})$ all exist in $D_{n-1}(i_j)$. In addition to the already existing 2-faces, there are new 2-faces in $D_{n-1}(i_j)$. Thus, $fs_2^{(\mathrm{block})}(i_j)$ can be divided into two parts as follows:

$$fs_2^{(\mathrm{block})}(i_j) = fs_2^{(\mathrm{block})}(i_{j-1})fs_2^{(\mathrm{new})}. \quad (18)$$

$fs_2^{(\mathrm{block})}(i_{j-1})$ can be determined from $D_{n-1}(i_{j-1})$. To determine $fs_2^{(\mathrm{block})}(i_j)$, we consider fs_2^{+} defined as

$$fs_2^{+}(i_j) = fs_2^{(\mathrm{block})}(i_j)f_2(N(i_{j-1})+1)f_2(N(i_{j-1})+2)$$

$$f_2\big(N(i_{j-1})+3\big)\cdots f_2(N_2). \qquad (19)$$

Here, $N(i_{j-1})$ is the number of 2-faces on $D_{n-1}(i_{j-1})\&D_n(i-1)$. By construction, the first Y digits of $fs_2^{+}(i_j)$ forms $fs_2^{(\text{block})}(i_j)$. Here, Y is the number of 2-faces on $D_{n-1}(i_j)$. However, we do not know Y beforehand. To find out $fs_2^{(\text{block})}(i_j)$, we decode $fs_2^{+}(i_j);pp$ using the $p_{n-1}^{(fs_2)}$-code until $D_{n-1}(i_j)$ is obtained. We count the number of 2-faces on $D_{n-1}(i_j)$. If there are $N(i_{j-1})+\beta$ 2-faces, then

$$fs_2^{(\text{block})}(i_j) = fs_2^{(\text{block})}(i_j) f_2\big(N(i_{j-1})+1\big) f_2\big(N(i_{j-1})+2\big)$$
$$f_2\big(N(i_{j-1})+3\big)\cdots f_2\big(N(i_{j-1})+\beta\big). \qquad (20)$$

If bpanel-na-pairs are found in decoding, record them in $pp(i_j)$. By putting $fs_2^{(\text{block})}(i_j)$ and $pp(i_j)$ together, it turns out to be $p_{n-1}^{(fs_2)}(i_j) = fs_2^{(\text{block})}(i_j);pp(i_j)$, from which we can determine $p_{n-2}(i_1)p_{n-2}(i_2)$ $p_{n-2}(i_3)\cdots p_{n-2}(i_j)$, thus $p_{n-2}(i_j)$.

To summarize, p_n can be recovered from $p_n^{(fs_2)} = fs_2;pp = f_2(1)f_2(2)f_2(3)$ $\cdots f_2(N_2);pp$ as follows (Supplementary Figure S1):

1. Determine $p_{n-1}(1)$ as follows:
   (a) Decode $f_2(1)f_2(2)f_2(3)\cdots f_2(N_2);pp$ using the the $p_{n-1}^{(fs_2)}$-code.
   (b) When an $(n-1)$-polytope is completed, count the number of 2-faces on the polytope. If there are α 2-faces on the polytope, then $fs_2^{(\text{block})}(1) = f_2(1)f_2(2)f_2(3)\cdots f_2(\alpha)$.
   (c) If bpanel-na-pairs are found in decoding, record them in $pp(1)$.
   (d) Determine $p_{n-1}(1)$ from $p_{n-1}^{(fs_2)}(1) = fs_2^{(\text{block})}(1);pp(1)$.

2. Determine the next $p_{n-1}(i) = ps_{n-2}(i); bp_{n-3}(i)$ $(2 \leq \mathrm{i})$ as follows:

   (a) $p_{n-2}(i_1) = \mathcal{M}[\mathrm{s-bpanel}]$.

   (b) To determine the next $p_{n-2}(i_j)$ $(2 \leq \mathrm{j})$, we examine pp. Here, two cases arise:

      (I) If the bpanel i_j is a w-bpanel, then $p_{n-2}(i_j) = \mathcal{M}[\mathrm{v-bpanel}]$.

      (II) Otherwise, we examine the k-joint, and then additional two cases arise:

         (i) If the k-joint is contributed by three blocks, then $p_{n-2}(i_j) = \mathcal{M}[\mathrm{c-panel}]$.

         (ii) Otherwise:

            (A) Decode $fs_2^{(\mathrm{block})}(i_{j-1})f_2(N(i_{j-1})+1)\cdots f_2(N_2); pp$ using the $p_{n-1}^{(fs_2)}$-code until $D_{n-1}(i_{j-1})$ is obtained.

            (B) Suppose that there are $N(i_{j-1}) + \beta$ 2-faces on $D_{n-1}(i_{j-1})$, then $fs_2^{(\mathrm{block})}(i_j) = fs_2^{(\mathrm{block})}(i_{j-1})f_2(N(i_{j-1})+1)\cdots f_2(N(i_{j-1})+\beta)$.

            (C) If bpanel-na-pairs are found in decoding, record them in $pp(i_j)$.

            (D) Determine $p_{n-2}(i_j)$ from $p_{n-1}^{(fs_2)}(i_j) = fs_2^{(\mathrm{block})}(i_j); pp(i_j)$.

   (c) Decode $p_{n-2}(i_1)p_{n-2}(i_2)p_{n-2}(i_3)\cdots p_{n-2}(i_j); pp$ using the p_{n-1}-code. Two cases then arise:

      (I) If an $(n-1)$-polytope is completed, then $ps_{n-2}(i) = p_{n-2}(i_1)p_{n-2}(i_2)$ $p_{n-2}(i_3)\cdots p_{n-2}(i_j)$. If bpanel-na-pairs are found, record their corresponding local side IDs in $bp_{n-3}(i)$. By putting $ps_{n-2}(i)$ and $bp_{n-3}(i)$ together, $p_{n-1}(i) = ps_{n-2}(i); bp_{n-3}(i)$ is obtained.

      (II) Otherwise, repeat the procedure 2b.

3. Decode $p_{n-1}(1)p_{n-1}(2)p_{n-1}(3)\cdots p_{n-1}(i);pp$ using the the p_n-code. Two cases then arise:

   (a) If an n-polytope is completed, then $ps_{n-1} = p_{n-1}(1)p_{n-1}(2)p_{n-1}(3)\cdots$ $p_{n-1}(i)$. If bpanel-na-pairs are found, record their corresponding local side IDs in bp_{n-2}. By putting ps_{n-1} and bp_{n-2} together, $p_n = ps_{n-1};bp_{n-2}$ is obtained.

   (b) Otherwise, repeat the procedure 2.

6. Unify $\boldsymbol{p_n^{(fs_2)}}$-representations into $\boldsymbol{p}$-representation. The subscript "n" of $p_n^{(fs_2)}$ indicates the dimension of the polytope represented by $p_n^{(fs_2)}$. This means the representation method for polytopes depends on the dimension of the polytope. But we can unify the $p_n^{(fs_2)}$-representations into the p-representation just by removing the subscript "n" and superscript "(fs_2)" from $p_n^{(fs_2)}$ to construct p. In decoding p, we do not know the dimension of the polytope beforehand. But we can determine the dimension as follows. We first decode p using the $p_3^{(fs_2)}$-code. If all the 2-faces are decoded when a 3-polytope is recovered, then p is $p_3^{(fs_2)}$. Otherwise, we decode p using the $p_4^{(fs_2)}$-code. If all the 2-faces are decoded when a 4-polytope is recovered, then p is $p_4^{(fs_2)}$. By repeating this procedure, the dimension of the polytope can be determined from p.
